# Plakophilin 2 gene therapy prevents and rescues arrhythmogenic right ventricular cardiomyopathy in a mouse model harboring patient genetics

**DOI:** 10.1038/s44161-023-00370-3

**Published:** 2023-12-07

**Authors:** William H. Bradford, Jing Zhang, Erika J. Gutierrez-Lara, Yan Liang, Aryanne Do, Tsui-Min Wang, Lena Nguyen, Nirosh Mataraarachchi, Jie Wang, Yusu Gu, Andrew McCulloch, Kirk L. Peterson, Farah Sheikh

**Affiliations:** 1 Department of Medicine, University of California San Diego, La Jolla, CA USA; 2 Department of Bioengineering, University of California San Diego, La Jolla, CA USA

**Keywords:** Cardiomyopathies, Genetics research, Genetic vectors

## Abstract

Arrhythmogenic right ventricular cardiomyopathy (ARVC) is a fatal genetic heart disease characterized by cardiac arrhythmias, in which fibrofatty deposition leads to heart failure, with no effective treatments. Plakophilin 2 (*PKP2*) is the most frequently mutated gene in ARVC, and although altered RNA splicing has been implicated, there are no models to study its effect and therapeutics. Here, we generate a mouse model harboring a *PKP2* mutation (IVS10-1G>C) affecting RNA splicing, recapitulating ARVC features and sudden death starting at 4 weeks. Administering AAV-PKP2 gene therapy (adeno-associated viral therapy to drive cardiac expression of *PKP2*) to neonatal mice restored PKP2 protein levels, completely preventing cardiac desmosomal and pathological deficits associated with ARVC, ensuring 100% survival of mice up to 6 months. Late-stage AAV-PKP2 administration rescued desmosomal protein deficits and reduced pathological deficits including improved cardiac function in adult mice, resulting in 100% survival up to 4 months. We suggest that AAV-PKP2 gene therapy holds promise for circumventing ARVC associated with *PKP2* mutations, including splice site mutations.

## Abstract (web summary)

Bradford, Zhang and colleagues generate a mouse model harboring a mutation that impacts *PKP2* splicing and show that one-time administration of AAV-PKP2 in neonatal mice could restore PKP2 and prevent the onset of key pathological features of arrhythmogenic right ventricular cardiomyopathy, and one-time administration in adult mice could rescue the phenotype and prevent sudden death up to 4 months post treatment.

## Main

ARVC is an incurable genetic heart disease that was first characterized as affecting only the right ventricle; however, increasing evidence highlights that there are left-dominant and biventricular forms of ARVC as well^1–3^. ARVC is classically characterized by early electrical defects, with a high frequency of ventricular arrhythmias that can be exacerbated with exercise, leading to sudden cardiac death^1,2^. However, structural defects are equally important, as ARVC progresses from an electrical phase to a structural phase in which there is fibrofatty replacement of the myocardium, leading to ventricular dysfunction and failure^1–3^. ARVC occurs in 1 out of 2,000–5,000 people, though its prevalence may be higher owing to poor diagnostic markers^1,4^. To date there are no effective treatments or cures for ARVC^5–7^. Current approaches are directed towards symptomatic relief and centered around lifestyle changes (for example, avoiding competitive sports that can trigger sudden cardiac death) and pharmacological interventions that target the electrical defects (for example, anti-arrhythmics drugs and beta-blockers), but also more invasive measures (for example, implantable cardioverter defibrillators, cardiac catheter ablation and heart transplantation), if the patient becomes intolerant or unresponsive to pharmacotherapy^5,7^. However, implantable cardioverter defibrillators have frequent device-related or lead-related complications, catheter ablations are subject to recurrence due to the generation of arrhythmogenic foci, and heart transplantation has a 23% mortality rate 10 years post procedure^7^. These factors highlight the critical need to identify therapeutic strategies that target the underlying drivers of the pathogenesis of ARVC.

Human genetic studies show that 40–50% of patients with ARVC carry mutations in genes of the desmosome^8^, which is a cell–cell adhesion structure that acts as a mechanical anchor and is therefore critical in tissues undergoing constant mechanical stress, such as cardiac muscle tissue^8,9^. Five classic genes make up the desmosome: desmoplakin (*DSP*), desmoglein 2 (*DSG2*), desmocollin 2 (*DSC2*), plakoglobin (*JUP*) and plakophilin 2 (*PKP2*), all of which have been implicated in human ARVC^7,8^. At the cardiac cell–cell junction, desmosomes function alongside the fascia adherens junction (which links the cell membrane to the actin cytoskeleton) and gap junctions (involved in electrical coupling) to coordinate muscle contraction^9^. Therefore, instrumental to understanding the pathogenesis of ARVC in the context of human genetics is that a single desmosomal gene mutation has a devastating ‘domino’ effect on the loss of expression of adjacent desmosomal proteins and neighboring gap junction proteins^7,10^. Although the neighboring fascia adherens junction proteins are initially spared, there is evidence that they can be affected in late stages of heart failure^10^, highlighting that ARVC is a disease of the desmosome with multi-protein consequences at the cell–cell junction.

*PKP2* mutations have been reported to make up the majority of desmosomal gene mutations in ARVC^7,8^. Mechanistically, studies of patients with ARVC suggest that a variety of *PKP2* mutations (insertions or deletions, nonsense, missense) lead to haploinsufficiency and therefore a reduction in myocardial PKP2 protein levels^11–13^. An effect on PKP2 RNA has also been reported, as myocardial *PKP2* transcript levels were also found to be downregulated in patients with ARVC harboring *PKP2* mutations^13,14^. More direct evidence of RNA involvement is suggested by studies that show that altered RNA splicing may also be a critical mechanism through which PKP2 patient genetics drive ARVC^11,15^, highlighting that altered PKP2 RNA levels may be a critical trigger of ARVC pathogenesis in patients with ARVC who have *PKP2* mutations. Defects in RNA splicing have been linked to approximately one-third of all human disease-causing mutations^16^, further highlighting the relevance of studying this mechanism in human ARVC. Splice acceptor sites are splicing elements located at the 3′ intron–5′ exon boundary and are essential for the excision of introns to generate a mature mRNA^17^. Mutations in these regions are linked to many human diseases, such as arthrogyrposis multiplex congenita, Charcot–Marie–Tooth disease and Fabry disease^17–20^. Splice acceptor site mutations trigger aberrantly spliced transcripts through various mechanisms, including the use of a cryptic intronic splice site, exon skipping or the use of a cryptic exonic splice site, resulting in diverse effects on protein quality and levels^15,18–20^. However, there are no models and limited mechanistic insights exist into how human mutations in RNA splicing affect PKP2 biology and ARVC, and into the therapeutics or interventions that would be most impactful.

Here, through the generation of a *PKP2* genetic mouse model, we highlight the sufficiency and molecular mechanisms by which a prevalent human *PKP2* RNA splice acceptor site mutation (*PKP2* IVS10-1G>C) triggers the postnatal onset of ARVC. *PKP2* homozygous mutant (Hom) hearts displayed altered *PKP2* RNA splicing, resulting in low levels of a higher molecular weight mutant PKP2 protein in the absence of endogenous PKP2 protein levels. These molecular consequences were sufficient to trigger an early and progressive disruption of the desmosome and development of all classic ARVC features (sudden cardiac death, arrhythmias, biventricular dysfunction and fibrofatty replacement of myocardium). In vitro studies showed that interventions focused on restoring PKP2 levels, irrespective of wild-type or mutant PKP2, were sufficient to prevent desmosomal protein disruption in *PKP2* Hom neonatal mouse cardiomyocytes. In vivo studies leveraging an adeno-associated viral (AAV) gene therapy strategy to drive cardiac expression of *PKP2* (AAV-PKP2) at early and late disease stages in *PKP2* Hom mice found that it was sufficient to restore PKP2 protein dose, scaffold the desmosome and prevent ARVC-related deficits, including prolonging lifespan. These data suggest that therapeutic approaches targeted at restoring PKP2 RNA and protein levels (via *PKP2* gene therapy) may have broad applicability to circumvent ARVC deficits associated with *PKP2* mutations, including splice site mutations.

## Results

### *PKP2* splice site mutation can recapitulate ARVC in mice

**Fig. 1:**
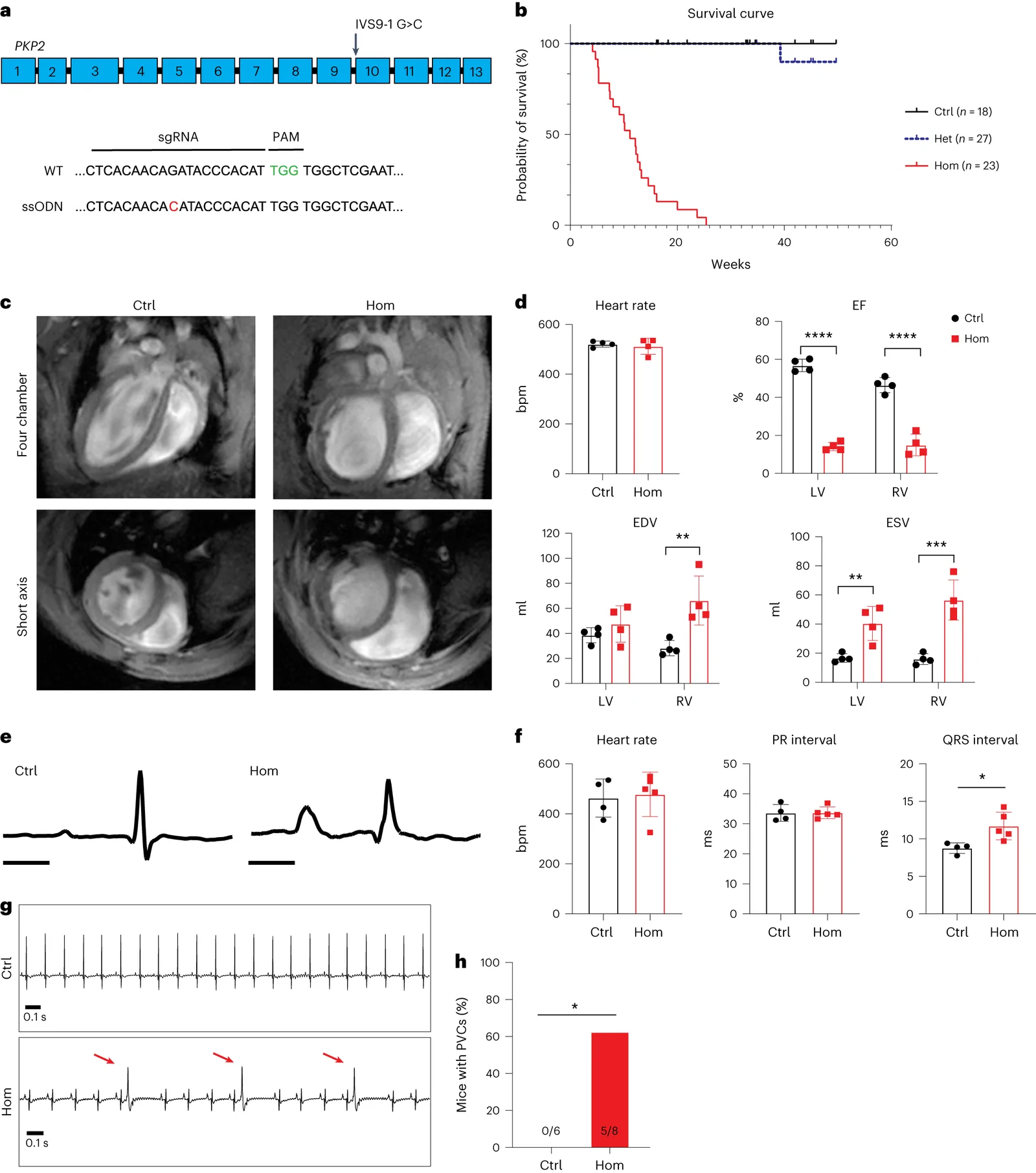
PKP2 splice site mutation is sufficient to recapitulate premature postnatal lethality associated with, and cardiac physiological disease hallmarks of ARVC in mice. **a**, Genomic location for *PKP2* IVS10-1G>C mutation equivalent in mouse (*PKP2* IVS9-1G>C), single strand oligodeoxynucleotides (ssODN) with mutation template (mutation is highlighted in red). PAM, protospacer adjacent motif, labeled in green; sgRNA, single guide RNA; WT, wild type. **b**, Kaplan–Meier survival analysis of *PKP2* Het, *PKP2* Hom and littermate control (Ctrl) mice. **c**, Representative cardiac four-chamber and short-axis views from MRI at end-diastole. **d**, Quantification of left (LV) and right (RV) ventricle end-systolic volume (ESV), end-diastolic volume (EDV), ejection fraction (EF) and heart rate using cine MRI in control (black bars) and *PKP2* Hom (red bars) mice (*n* = 4 biologically independent animals). Data are presented as mean ± s.e.m. Two-way analysis of variance (ANOVA) with Bonferroni’s multiple comparison test. Adjusted *P* values, ****P < 0.00001, **P < 0.01 (EDV, RV, *P* = 0.0026; ESV, LV, *P* = 0.0074), ***P < 0.001 (ESV, RV, *P* = 0.0001). **e**, Representative composite surface ECG tracings averaged from four beats in control and *PKP2* Hom mice at 4 weeks of age. Scale bars, 10 ms. **f**, Quantification of heart rate, PR interval and QRS interval from composite surface ECG tracings (controls, *n* = 4; Hom, *n* = 5 biologically independent animals). Data are presented as mean ± s.e.m. Two-tailed unpaired *t*-test. *P < 0.05 (*P* = 0.0198). **g**, Representative ECG tracings from control and *PKP2* Hom mice at 4 weeks of age (controls, *n* = 6; Hom, *n* = 8 biologically independent animals). **h**, Quantification of mice demonstrating PVCs (right, red arrows) (controls, *n* = 6; Hom, *n* = 8 biologically independent animals). Two-tailed Fisher’s exact test. *P < 0.05 (*P* = 0.0310). Source data

**Extended Data Fig. 1:**
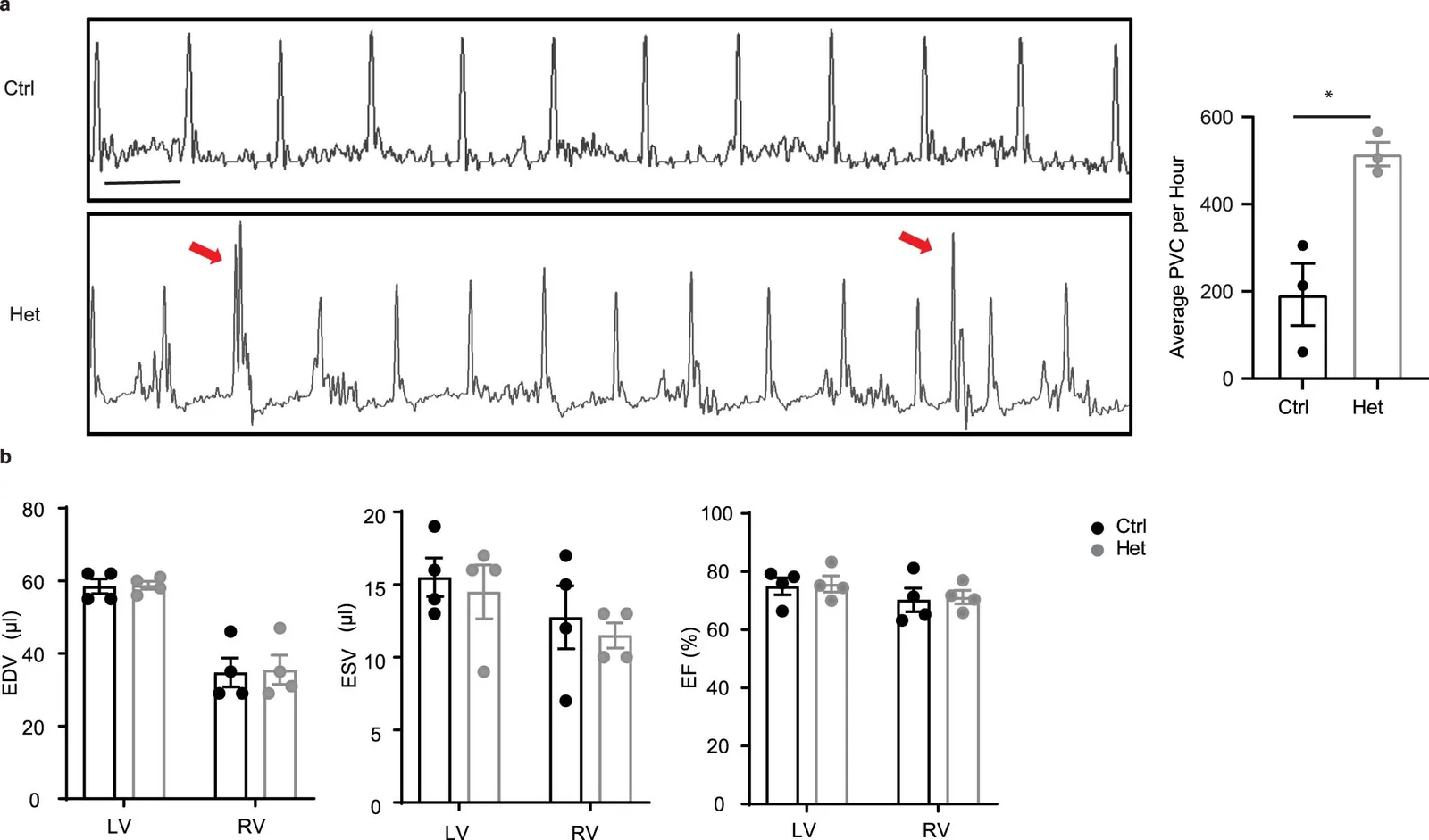
Telemetry electrocardiogram (ECG) and cardiac magnetic resonance image (MRI) analysis in PKP2 Het mice. (**a**) Representative telemetry ECG tracings and premature ventricular contraction (PVC) counts from Ctrl and PKP2 heterozygous (Het) mice at 3 months of age. Red arrows indicate premature ventricular contractions. Scale bar = 0.1 s. Data are presented as mean ± S.E.M. n = 3 biologically independent animals examined per group, two-tailed unpaired t test, * p < 0.05 (p = 0.0135). (**b**) Quantification of end-diastolic volumes (EDV), end-systolic volumes (ESV) and percentage of ejection fraction (EF %) in WT and PKP2 Het mice at 10 months old of age using cardiac MRI. n = 4 biologically independent animals examined per group. Data are presented as mean ± S.E.M. Two-way ANOVA with Tukey’s multiple comparison test.

Previous studies identified a *PKP2* RNA splice acceptor site mutation (*PKP2* IVS10-1G>C) in multiple ARVC populations in which patients displayed classic ARVC disease features^11,15,21,22^, contributing to the idea that alterations in *PKP2* RNA splicing may be a critical mechanism through which *PKP2* patient genetics drive ARVC. This mutation is also highly relevant as it ranks at the top of the list of most frequent *PKP2* mutations in ARVC cohorts in the Atlas of Cardiac Genetic Variation^23^. To investigate the direct consequence of the *PKP2* RNA splice site IVS10-1G>C mutation in the pathogenesis of ARVC, we created a knock-in mouse model within mouse intron 9 (mouse IVS9-1G>C equivalent to human IVS10-1G>C) through CRISPR–Cas9 genome editing (Fig. 1a). Founder mice harboring the *PKP2* IVS10-1G>C mutation were backcrossed three generations to remove CRISPR–Cas9-associated off-target effects and generate *PKP2* IVS10-1G>C heterozygous (Het) mutant mice. All offspring were viable and born at Mendelian ratios (Supplementary Table 1); however, *PKP2* IVS10-1G>C Hom mice displayed sudden death beginning at 4 weeks of age, with a median survival of 11 weeks, and no *PKP2* Hom mice survived past 26 weeks of age (Fig. 1b). *PKP2* Het mice also showed survival defects, although not as severe as those of *PKP2* Hom mice (Fig. 1b). Telemetry electrocardiogram (ECG) recordings revealed that conscious *PKP2* Het mice displayed baseline electrophysiological abnormalities (marked by the presence of premature ventricular contractions (PVCs)) in the absence of cardiomyopathy, as assessed by in vivo magnetic resonance imaging (MRI), which may account for premature lethality and sudden cardiac death at later ages (Extended Data Fig. 1). To better understand the onset of sudden cardiac death in *PKP2* Hom mice (4 weeks of age), we quantitatively assessed cardiac dimensions and function in the left and right ventricles of 4-week-old *PKP2* Hom mice and littermate controls using MRI. Representative four-chamber and short-axis views highlighted biventricular dilation in *PKP2* Hom mice compared to littermate controls (Fig. 1c). No significant differences in heart rate were observed between mice (Fig. 1d); however, a significant decrease in left and right ventricular ejection fraction was found in *PKP2* Hom mice compared to controls (Fig. 1d). Defects in cardiac function were attributed to the significantly enlarged right ventricular end-diastolic and end-systolic volumes (Fig. 1d), as well as significantly increased left ventricular end-systolic volumes in *PKP2* Hom mice (Fig. 1d), highlighting biventricular dysfunction. These results are reminiscent of those in patients harboring the *PKP2* IVS10-1G>C mutation, in whom both left and right ventricular dysfunction were observed^21^.

**Extended Data Fig. 2:**
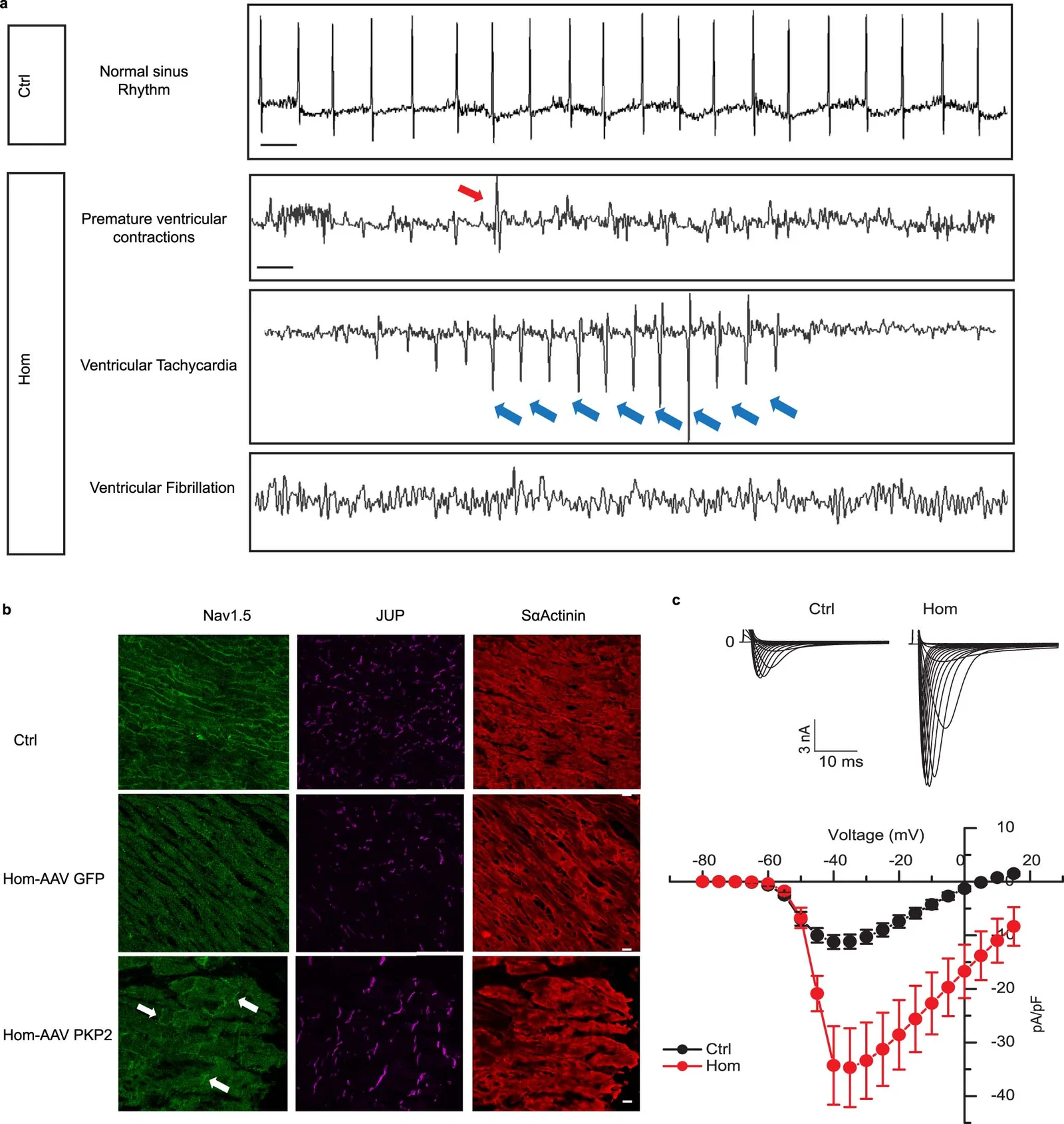
PKP2 Hom mice display various electrophysiological abnormalities that precede premature death. (**a**) Representative telemetry ECG tracings from Ctrl and PKP2 Hom (Hom) mice. Note that PKP2 Hom mice display isolated PVCs, that transition to ventricular tachycardia and fibrillation. Red arrow indicates PVC; Blue arrows indicate ventricular tachycardia. Scale bar = 0.1 s. (**b**) Immunofluorescence staining of Nav1.5, plakoglobin (JUP), and the cardiomyocyte marker, sarcomeric alpha-actinin in representative mouse heart sections from 9 week old Ctrl, PKP2 Hom + AAV-GFP (five weeks post-late stage administration) and PKP2 Hom+AAV-PKP2 (five weeks post-late stage administration). Scale bar, 10 μm. White arrows depict Nav1.5 re-localization to cell-cell junction/membrane in PKP2 Hom hearts. Experiments were repeated independently three times with similar results. (**c**) Representative sodium current recording (left) and ion current-voltage (I-Vm) relationship of sodium current (right) in isolated adult cardiomyocytes from 8-9 week old Ctrl and PKP2 Hom mice, highlighting loss of sodium channel homeostasis in PKP2 Hom cardiomyocytes. Current amplitude has been normalized against cell capacitance (pA/pF). Data are presented as mean ± S.E.M. Ctrl, n = 6 biologically independent animals; PKP2 Hom, n = 8 biologically independent animals. Two-way ANOVA, **** p < 0.0001.

Electrical dysfunction is a key hallmark of ARVC and a major contributor to sudden cardiac death in patients^2^. To assess electrical defects in *PKP2* Hom mice, we performed surface ECGs in 4-week-old mice. The analysis of composite surface ECG tracings from *PKP2* Hom mice and littermate controls showed no difference in heart rate or PR intervals; however, QRS complexes were significantly widened in *PKP2* Hom mice (Fig. 1e,f), indicative of ventricular depolarization abnormalities, which are common in patients with ARVC^1–3^. Ventricular depolarization abnormalities can serve as a primer for ventricular re-entry that is termed PVC^24^. The analysis of surface ECG tracings revealed PVCs in 60% of *PKP2* Hom mice at 4 weeks of age (onset of sudden death), whereas none were observed in littermate controls (Fig. 1g,h). Telemetry ECG recordings revealed that conscious *PKP2* Hom mice had isolated PVCs that precipitated into ventricular tachycardia and fibrillation (Extended Data Fig. 2), further suggesting that ventricular depolarization abnormalities can serve as a primer for life-threatening arrhythmias. *PKP2* has also been implicated in driving alterations to sodium channel current homeostasis and Nav1.5 localization or expression in ARVC^25,26^. Whole-cell patch-clamp electrophysiological analysis of adult *PKP2* Hom cardiomyocytes revealed alterations in sodium current homeostasis compared to that of controls (Extended Data Fig. 2). Immunofluorescence microscopy also showed loss of Nav1.5 cell–cell junction and membrane protein localization in *PKP2* Hom cardiomyocytes when compared with controls (Extended Data Fig. 2). Together, these data suggest that *PKP2* Hom mice recapitulate key electrical disease features found to be characteristic of ARVC.

**Fig. 2:**
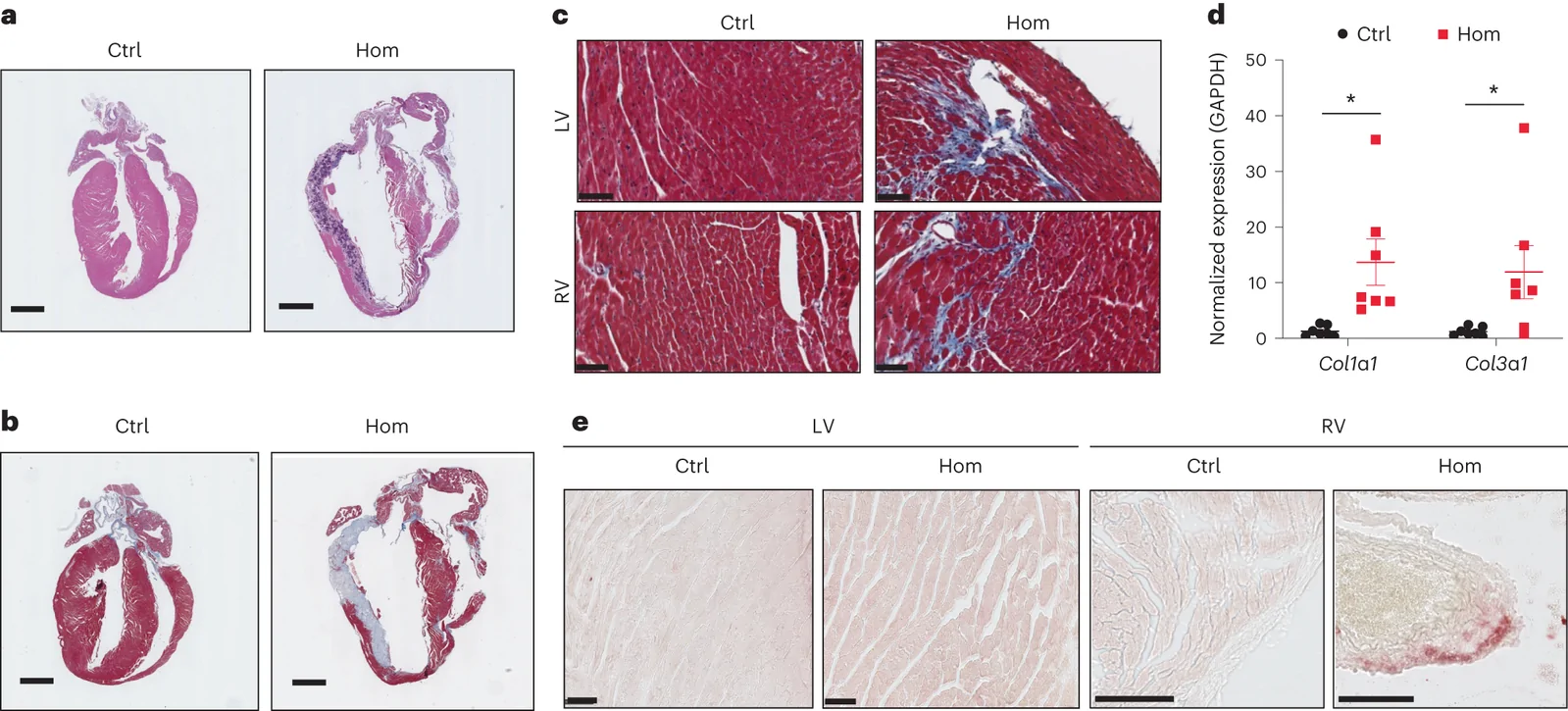
PKP2 splice site mutation is sufficient to recapitulate cardiac pathological disease hallmarks associated with ARVC in mice. **a**,**b**, Representative cardiac sections stained with hematoxylin and eosin (**a**) and Masson’s Trichrome staining (**b**) from control and *PKP2* Hom mice at 6 weeks of age. Scale bars, 1 mm. **c**, High-magnification views of Masson’s Trichrome stained sections from the left ventricle and right ventricle. Scale bars, 50 µm. **d**, Quantification of fibrosis with RT–qPCR for profibrotic gene markers *Col1α1* and *Col3α1* (controls, *n* = 5; Hom, *n* = 7 biologically independent animals). Data are presented as mean ± s.e.m. Two-tailed unpaired *t*-test. *P < 0.05 (*P* = 0.0116 for *Col1α1* and *P* = 0.0448 for *Col3α1*). **e**, Right ventricle and left ventricle sections stained with Oil Red O from control and *PKP2* Hom hearts at 6 weeks of age. Scale bars, 100 µm. Experiments were repeated independently three times with similar results.

**Extended Data Fig. 3:**
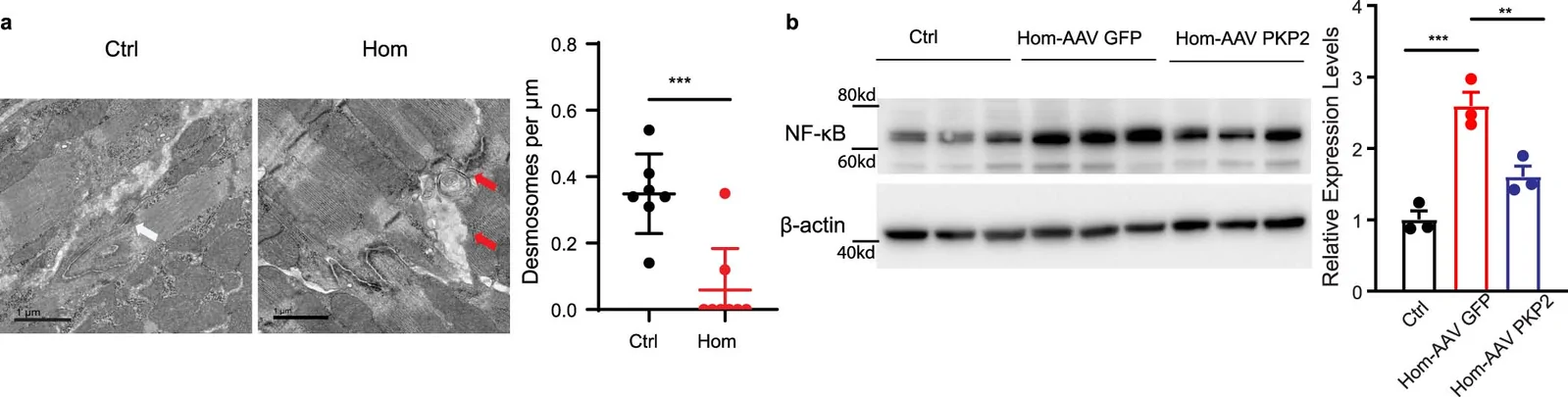
PKP2 Hom mice display cardiac ultrastructural and inflammatory deficits. (**a**) Representative transmission electron micrographs from Ctrl and PKP2 Hom hearts at 4 weeks of age. White arrows denote healthy desmosomes. Quantification of desmosomes per μm of membrane in Ctrl and PKP2 Hom hearts at two weeks of age, denoted early loss of desmosomes in PKP2 Hom. Red arrows denote multi-membrane vesicles, suggestive of desmosomal structure degradation. Scale bar = 1 µm. Data are presented as mean ± S.E.M. two-tailed t test, n = 7 for Ctrl, n = 8 for PKP2 Hom biologically independent animals per group, respectively. ***, p < 0.001 (p = 0.0005). (**b**) Representative western blot and quantification analysis of nuclear factor-kappa B (NF-κB) expression in heart lysates from 9 week old Ctrl, PKP2 Hom + AAV-GFP (five weeks post-late stage administration) and PKP2 Hom+AAV-PKP2 (five weeks post-late stage administration) mice. β-actin used as a loading control. Data are presented as mean ± S.E.M. One-way ANOVA with Turkey’s multiple comparisons test, n = 3 biologically independent animals per group, **, p < 0.01 (p = 0.0099), ***, p < 0.001 (p = 0.0009). Source data

**Extended Data Fig. 4:**
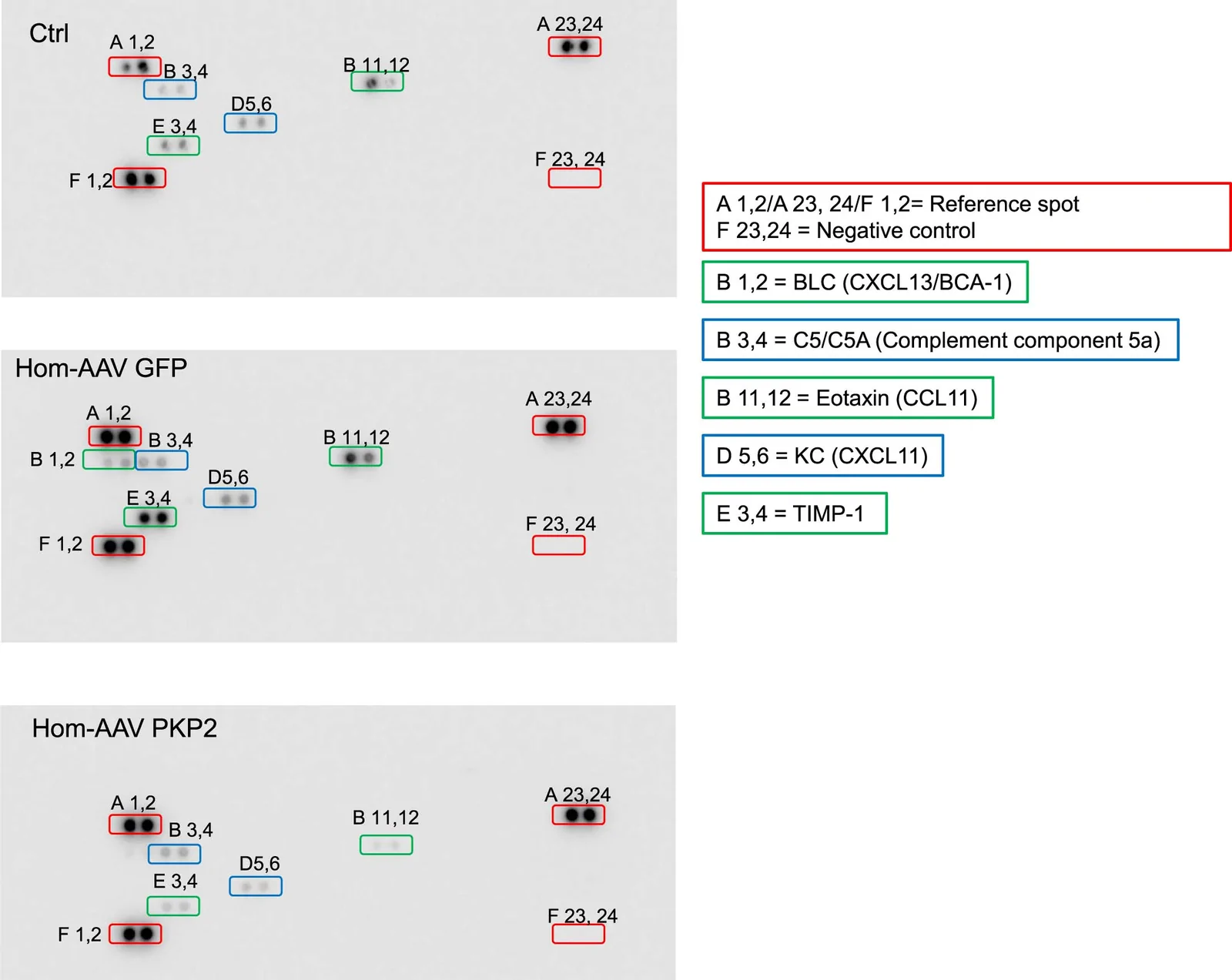
PKP2 Hom mice display blood serum cytokine alterations that can be alleviated with late-stage administration of AAV PKP2. Representative cytokine arrays are shown from serum collected from 9 weeks old Ctrl, PKP2 Hom + AAV-GFP (five weeks post-late stage administration) and PKP2 Hom+AAV-PKP2 (five weeks post-late stage administration) mice. Spots in red refer to reference markers to compare overall exposure levels. Spots in blue refer to cytokines (CXCL11) and C5/C5A (Complement component 5a) that are not significantly different between groups. Spots in green refer to cytokines (BLC (CXCL13/BCA-1); Eotaxin (CCL11); TIMP-1) that are altered in PKP2 Hom treated with AAV GFP and rescued by AAV PKP2 treatment in PKP2 Hom mice. Blood serum samples from Ctrl (n = 4 biologically independent animals), Hom-AAV GFP (n = 5 biologically independent animals) and Hom-AAV PKP2 (n = 6 biologically independent animals) were pooled for the cytokines array analysis.

Structural defects are also observed in human ARVC; these are desmosomal ultrastructural deficits and fibrofatty replacement of the myocardium^3,27^. Transmission electron microscopy analysis of *PKP2* Hom hearts at 4 weeks of age revealed enlarged gaps and an accumulation of multi-membrane vesicles at the cardiac cell–cell junction (Extended Data Fig. 3), indicative of protein degradation (protein degradation machinery accumulation) at this level. A similar phenomenon was observed in hearts deficient of constitutive photomorphogenesis 9 signalosome subunit 6 (CSN6), a desmosomal resident protein that, when lost, drives desmosomal protein degradation and ARVC^28^. Quantitative analysis of electron-dense desmosomes at the cell–cell junction revealed significantly reduced levels of desmosomes in *PKP2* Hom hearts as early as 2 weeks of age compared to controls (Extended Data Fig. 3), suggesting early postnatal loss of desmosomes in *PKP2* Hom hearts. To assess whether *PKP2* Hom mice recapitulate fibrofatty replacement of myocardium, cardiac histological analysis was performed at 6 weeks of age. Hematoxylin and eosin staining revealed extensive dilation and thinning of both left and right ventricles in *PKP2* Hom mice compared to littermate controls (Fig. 2a). Masson’s Trichrome staining highlighted severe loss of myocardium and fibrosis in both left and right ventricles (Fig. 2b,c). Quantitative PCR with reverse transcription (RT–qPCR) analysis validated the findings at the histological level, showing a significant increase in the expression of profibrotic genes *Col1a1* and *Col3a1* in *PKP2* Hom mice compared to littermate controls (Fig. 2d). Studies in patients with ARVC and mouse models of ARVC have demonstrated a particular affinity for fat deposition to more specifically localize in the subepicardium of the right ventricle, which represents a critical location to understand the mechanisms of adipogenesis in ARVC^24,29,30^. Using Oil Red O staining as a marker for neutral lipids, we show that 6-week-old *PKP2* Hom mouse hearts demonstrate lipid accumulation in the subepicardium of the right ventricle (Fig. 2e), a finding that was not observed in the left ventricles of *PKP2* Hom mice and hearts of wild-type littermate control mice (Fig. 2e). Thus, *PKP2* Hom mice display fibrofatty replacement of myocardium, a key hallmark of human ARVC. Recent evidence indicates that inflammatory pathways, such as cardiac NF-κB signaling, as well as secretory cytokine production are altered in ARVC settings^31^. We show that cardiac NF-κB protein expression is significantly upregulated in *PKP2* Hom hearts compared to controls, and that cytokine array profile analyses reveal a significant upregulation of specific cardiac inflammatory cytokines in the serum of *PKP2* Hom mice compared to controls (Extended Data Figs. 3 and 4). Together, these findings show the sufficiency of the *PKP2* IVS10-1G>C mutation to recapitulate the postnatal onset of key human ARVC disease features in *PKP2* Hom mice.

### *PKP2* mutation affects cardiac PKP2 quality and quantity

**Fig. 3:**
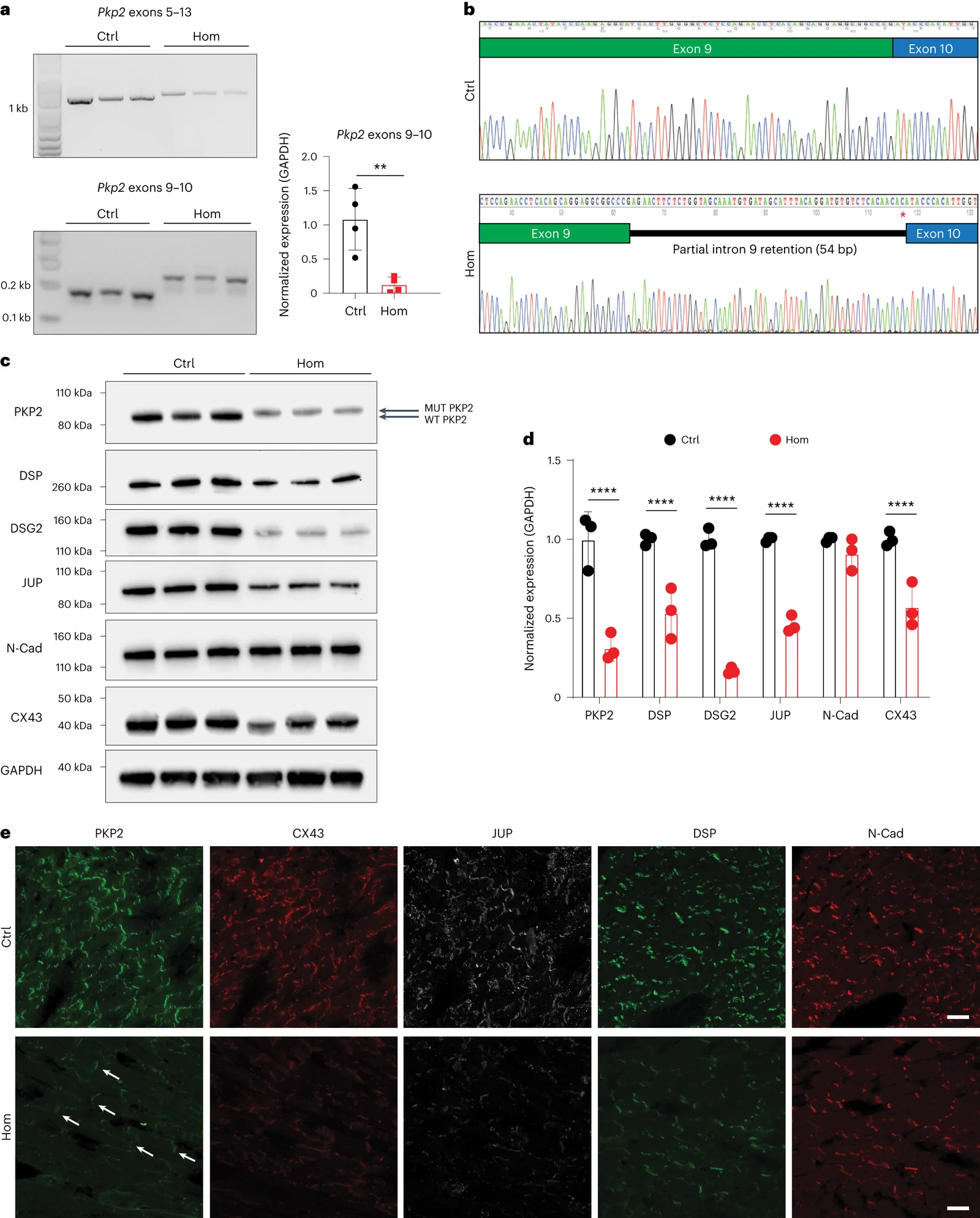
*PKP2* splice site mutation affects PKP2 quality (appearance of mutant PKP2) and reduces PKP2 levels with consequences on desmosomal and gap junction disruption in *PKP2* Hom mouse hearts. **a**, RT–qPCR analysis of *PKP2* exons 5–13 and exons 9–10 in control and *PKP2* Hom hearts at 4 weeks of age. RT–qPCR analysis of *Pkp2* exons 9–10 in control and *PKP2* Hom hearts (*n* = 4 biologically independent animals). Data are presented as mean ± s.e.m. Two-tailed unpaired *t*-test, **P < 0.01 (*P* = 0.0063). **b**, Sequencing analysis of RT–qPCR products from control and *PKP2* Hom hearts. Red asterisk denotes mutation site. **c**, Western blot analysis of desmosomal (PKP2, DSP and DSG2), fascia adherens (JUP and N-Cad) and gap junction (CX43) proteins at 4 weeks of age in control and *PKP2* Hom hearts. GAPDH served as the loading control. Endogenous WT and MUT PKP2 protein bands are depicted by black arrows. **d**, Quantification of protein expression in **a** normalized to GAPDH (*n* = 3 biologically independent animals). Experiments were repeated independently three times with similar results. Data are presented as mean ± s.e.m. Two-way ANOVA with Sidak’s multiple comparison test. ****P < 0.0001. **e**, Immunofluorescence staining of desmosomal, fascia adherens and gap junction proteins at 4 weeks of age in control and *PKP2* Hom hearts. Scale bars, 25 µm. White arrows indicate the localization of mutant PKP2 in *PKP2* Hom mice hearts. Experiments were repeated independently three times with similar results. Source data

To determine the molecular consequence of the *PKP2* IVS10-1G>C mutation on *PKP2* RNA, ventricular RNA was isolated from *PKP2* Hom and littermate control hearts at 4 weeks of age. RT–qPCR analysis of mouse *PKP2* exons 5–13 revealed a single, larger product in *PKP2* Hom hearts at what appeared to be reduced levels of endogenous PKP2 in control hearts (Fig. 3a). A more focused RT–qPCR analysis of the mutation locus using *PKP2* exons 9–10 primers similarly revealed a larger mutant product in *PKP2* Hom hearts in the absence of an endogenous PKP2 product (Fig. 3a). RT–qPCR analysis of *PKP2* exons 9–10 demonstrated a significant reduction in *PKP2* transcript levels in *PKP2* Hom hearts (Fig. 3a), suggesting that the *PKP2* IVS10-1G>C splice acceptor site mutation affected total *PKP2* RNA levels. Sanger sequencing analyses demonstrated contiguous sequences between *PKP2* exons 9 and 10 in control hearts, suggestive of splicing using the canonical intron 9 splice acceptor site (Fig. 3b). However, sequencing analyses of *PKP2* Hom hearts revealed an additional 54 base pairs between exons 9 and 10 (Fig. 3b), corresponding to the use of an alternative upstream splice site and suggestive of intron retention as a mechanism driving the larger *PKP2* transcript size.

To determine the effect of the *PKP2* IVS10-1G>C mutation on PKP2 and cardiac cell–cell junction protein homeostasis, western blot analysis was performed in ventricular lysates from *PKP2* Hom hearts and littermate controls at 4 weeks of age. A higher molecular weight mutant PKP2 protein was identified in the absence of endogenous PKP2 in *PKP2* Hom hearts (Fig. 3c). The mutant PKP2 protein was found at significantly reduced levels compared to wild-type PKP2 in littermate control hearts (Fig. 3c,d), suggesting the translation of the larger sized *PKP2* transcript into a higher molecular weight mutant PKP2 protein (Fig. 3c). These molecular consequences resulted in the direct loss of neighboring desmosomal components, including DSP, DSG2 and JUP, in *PKP2* Hom hearts (Fig. 3c,d). The molecular alterations at the desmosome had direct consequences on the predominant ventricular gap junction protein, CX43, which was significantly reduced in *PKP2* Hom hearts compared to controls (Fig. 3c–e). CX43 is thought to be a direct target of DSP, suggesting a molecular consequence of desmosomal disruption^24^. At this time point, levels of the fascia adherens marker N-Cad in *PKP2* Hom hearts were not significantly different from those of controls (Fig. 3c,d). Using the cell–cell junction marker N-Cad, immunofluorescence microscopy analyses showed that the mutant PKP2 as well as CX43, JUP and DSP were localized at the cell–cell junction in *PKP2* Hom hearts, albeit at reduced levels compared to those in control hearts (Fig. 3e), which is consistent with findings from western blot analysis (Fig. 3c,d). Together, these data demonstrate that the *PKP2* IVS10-1G>C splice acceptor site mutation affects PKP2 protein quality and quantity by the appearance of a higher molecular weight mutant PKP2 protein that seems to retain cell–cell junction localization, and by the loss of endogenous PKP2 protein, which results in reduced total PKP2 protein levels compared to littermate controls.

### PKP2 quantity deficits drive desmosomal protein loss

**Fig. 4:**
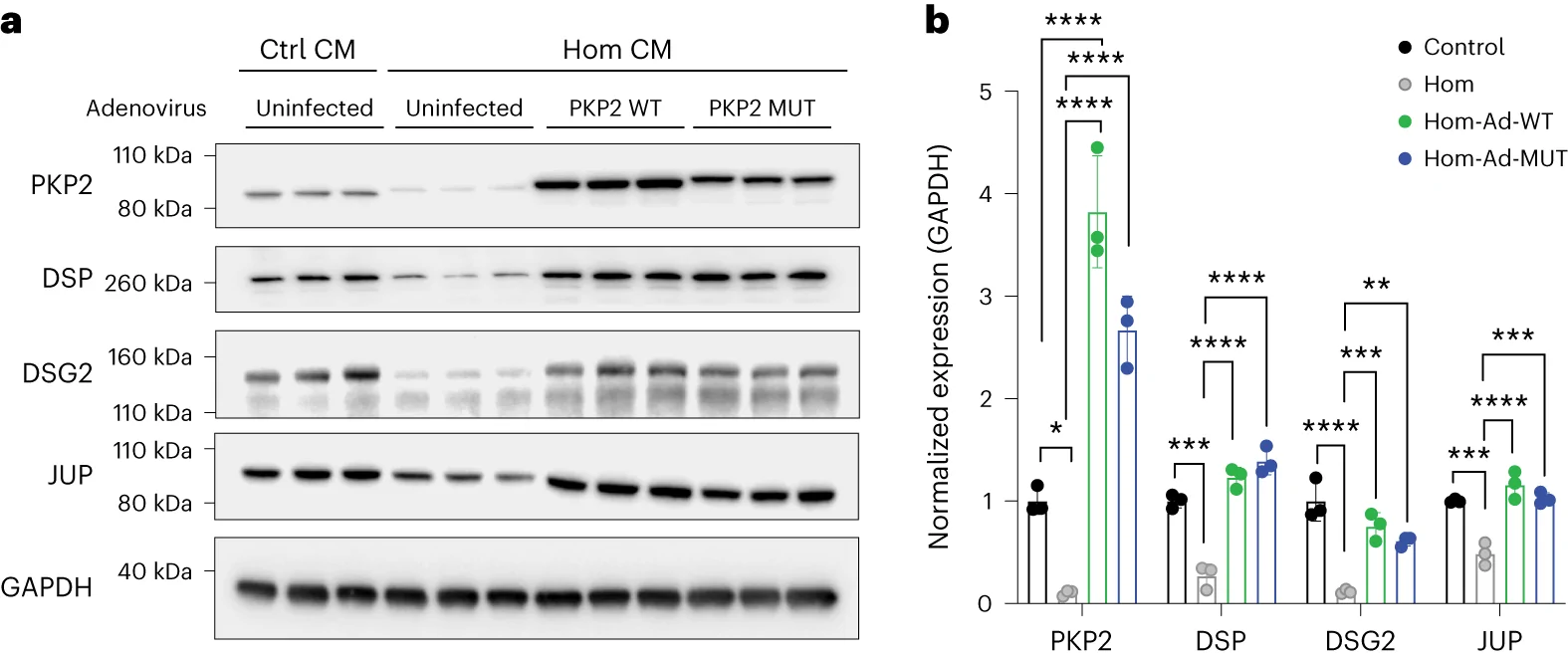
Increasing wild-type and mutant PKP2 protein levels rescues desmosomal protein loss in *PKP2* Hom neonatal cardiomyocytes in vitro. **a**, Representative western blot analysis of desmosomal (PKP2, DSP, DSG2, JUP) and fascia adherens junction (N-Cad) proteins in control and Hom cardiomyocytes infected with PKP2 WT and PKP2 MUT adenoviruses. Uninfected cells were used as a control. The experiments were repeated using five independent preparations of cells. Western blot analysis of GAPDH was used as a loading control. CM, cardiomyocyte. **b**, Quantification of western blot analysis. Data are presented as mean ± s.e.m., *n* = 3 individual experiments. One-way ANOVA with Tukey’s multiple comparison test was used to compare the significance among the treatments for the same protein. *P < 0.05 (PKP2, *P* = 0.0408), **P < 0.01 (DSG2, *P* = 0.0047), ***P < 0.001 (DSP, *P* = 0.0001; DSG2, *P* = 0.0009; JUP, *P* = 0.0004), ****P < 0.0001. Source data

**Extended Data Fig. 5:**
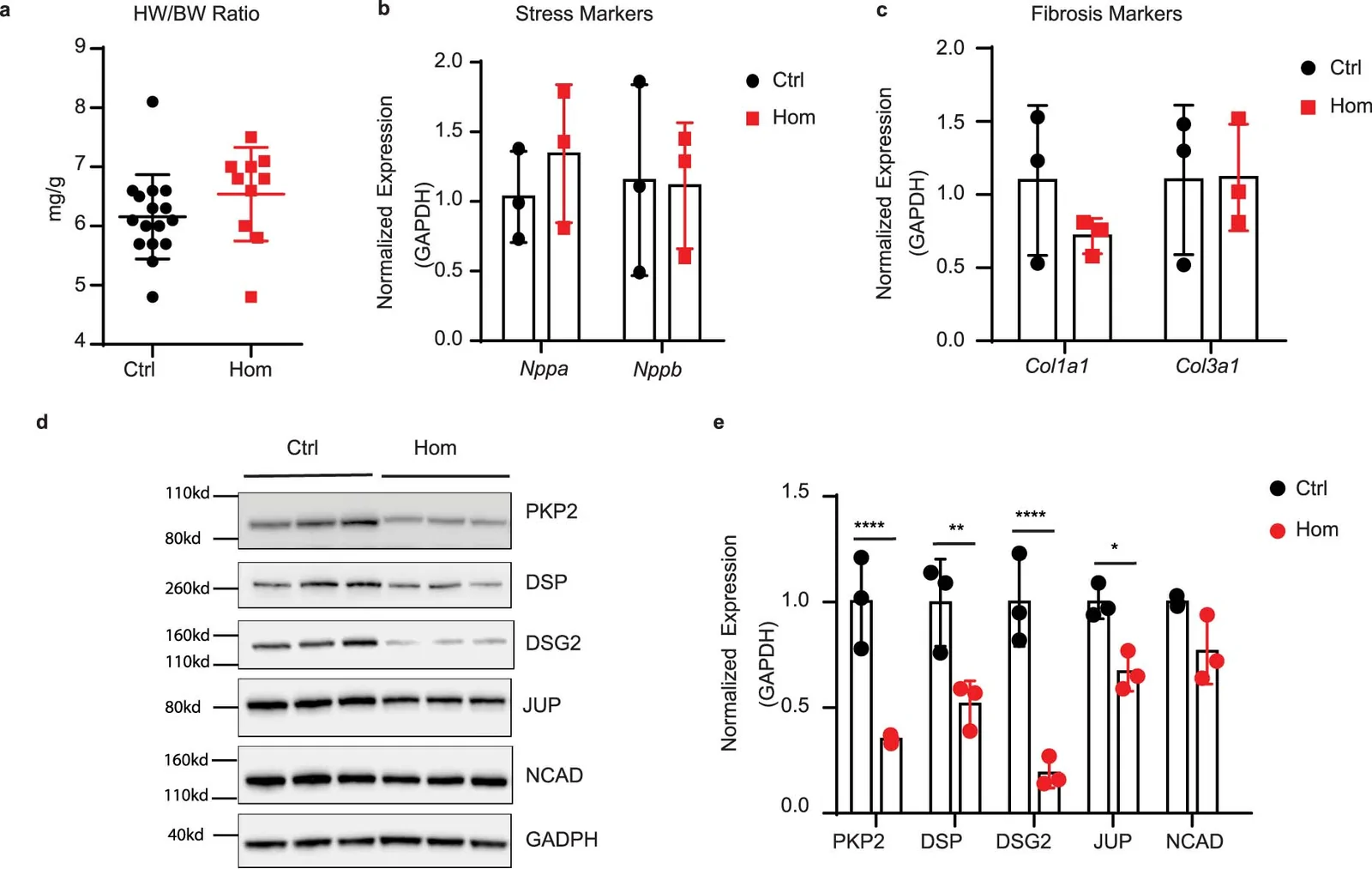
PKP2 Hom neonatal (postnatal day 1, P1) hearts display desmosomal deficits in the absence of gross morphological changes to the heart. **(a)** P1 heart weight to body weight ratios (HW/BW). Data are presented as mean ± S.E.M. n = 16 for Ctrl, n = 10 for Hom biologically independent animals, two-tailed unpaired t test, p = 0.2129, not significant. **(b)** RT-qPCR of cardiac stress markers atrial natriuretic factor (*Nppa*) and B-type natriuretic factor (*Nppb*) as well as **(c)** profibrotic genes collagen α1 types I (*Col1α1*) and III (*Col3 α1*) in P1 hearts. Data are presented as mean ± S.E.M. For **(b)** and **(c)**, n = 3 biologically independent animals per group, two-tailed unpaired t test between groups, p = 0.4171 (*Nppa*), p = 0.9368 (*Nppb*), p = 0.2800 (Col1 *α* 1), p = 0.9655 (*Col3 α* 1), not significant. **(d)** Western blot analysis of desmosomal (PKP2, DSP, DSG2, JUP) and fascia adherens junction (JUP, N-Cad) proteins in neonatal cardiomyocytes from Ctrl and PKP2 Hom (P1) hearts. Glyceraldehyde-3-Phosphate Dehydrogenase (GAPDH) serves as the loading control. **(e)** Quantification of protein expression in (d) normalized to GAPDH (n = 3 biologically independent animals per group). Data are presented as mean ± S.E.M. Two-way ANOVA with Tukey’s multiple comparison test. ****, p < 0.0001. **, p < 0.01 (p = 0.0011). *, p < 0.05 (p = 0.0335). Source data

To determine whether the early postnatal desmosomal disruption in *PKP2* Hom hearts is driven by alterations in PKP2 quality (that is, the toxicity of mutant PKP2) or quantity (that is, reduced protein levels compared to wild-type PKP2), we generated adenovirus vectors expressing wild-type (Ad WT) and mutant (Ad MUT) mouse *PKP2* to determine their effects on desmosomal protein levels in *PKP2* Hom neonatal mouse cardiomyocytes (Fig. 4a). At postnatal day 1–2, *PKP2* Hom mice do not show overt ARVC disease features, yet display ARVC-related molecular alterations, including disruption or loss of components of the desmosome (Extended Data Fig. 5). We observed a significant reduction in the desmosomal components PKP2 (endogenous), DSP, DSG2 and JUP, as well as appearance of a higher molecular weight PKP2 in uninfected *PKP2* Hom neonatal cardiomyocytes compared to control cardiomyocytes (Fig. 4). However, Ad PKP2 WT-treated *PKP2* Hom neonatal cardiomyocytes significantly increased DSP, DSG2 and JUP expression in *PKP2* Hom neonatal cardiomyocytes compared to uninfected *PKP2* Hom cardiomyocytes (Fig. 4). Interestingly, *PKP2* Hom neonatal cardiomyocytes treated with Ad PKP2 MUT also demonstrated a significant increase in DSP, DSG2 and JUP protein expression compared to uninfected *PKP2* Hom cardiomyocytes (Fig. 4). Given that the restoration of either wild-type or mutant PKP2 protein was sufficient to increase desmosomal protein levels, these data highlight that loss of PKP2 protein quantity and not quality (that is, mutant form) is the driver of desmosomal disruption in *PKP2* Hom neonatal cardiomyocytes and early ARVC. Furthermore, these findings show the ability of PKP2 to function as a potent molecular scaffold to restore near-wild-type expression levels of proteins at the desmosomal cell–cell junction.

### Early PKP2 restoration prevents ARVC deficits in mice

**Fig. 5:**
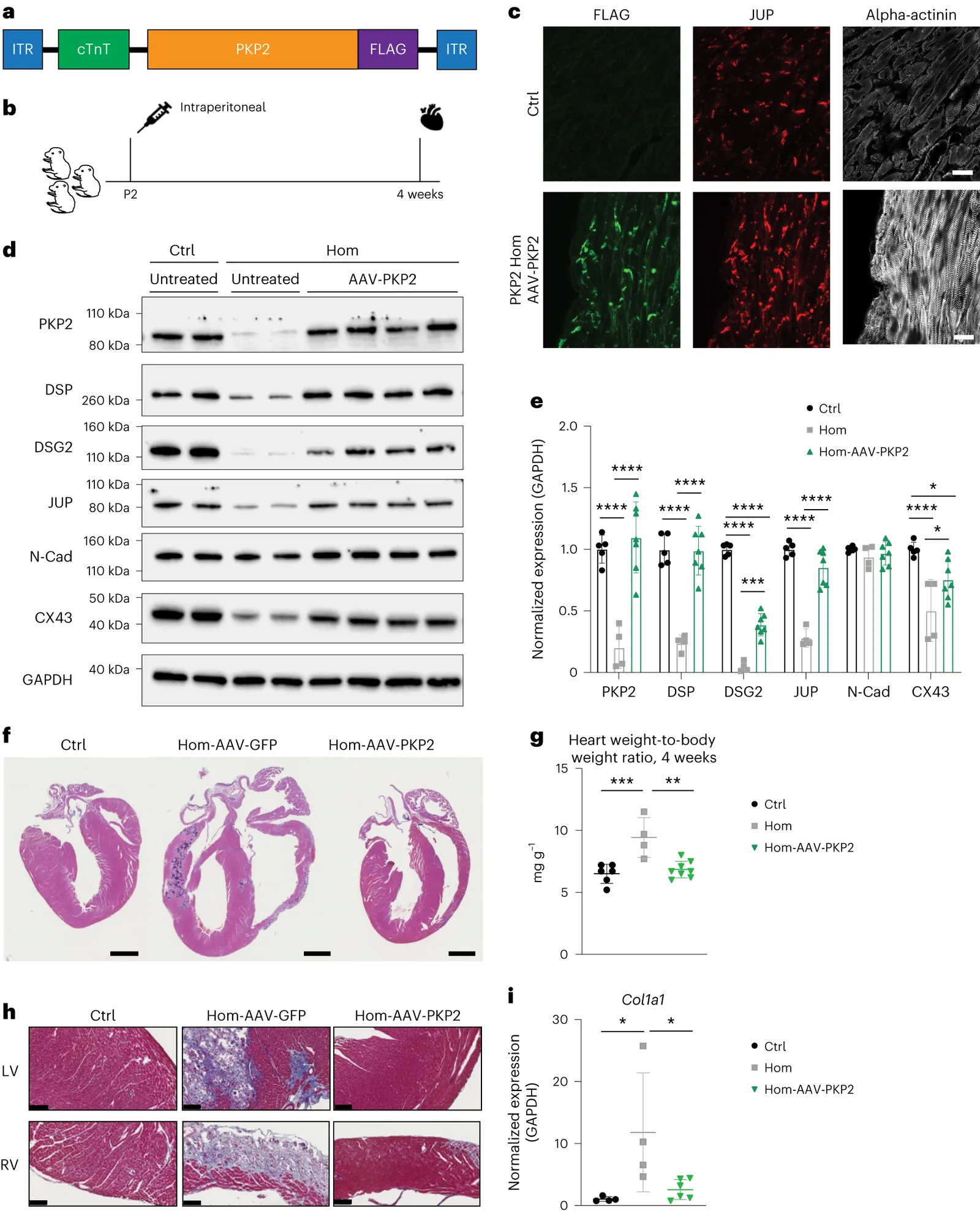
Early administration of AAV PKP2 prevents desmosomal-mediated cell junction deficits and cardiac pathological hallmarks of ARVC in *PKP2* Hom mice. **a**, Design of AAV *Pkp2* vector with inverted terminal repeats (ITR), cTnT promoter, Kozak sequence, *Pkp2* cDNA, C-terminal FLAG tag, Woodchuck hepatitis virus post-transcriptional regulatory element (WPRE) and bovine growth hormone polyadenylation signal (BGH-pA). **b**, Schemata of early neonatal delivery strategy and analysis time point. **c**, Representative immunofluorescence staining of FLAG (green), JUP (red) and alpha-actinin (white) at 4 weeks post injection in uninfected wild-type control and *PKP2* Hom hearts treated with AAV-PKP2. Scale bars, 20 µm. Experiments were repeated independently three times with similar results. **d**, Western blot analysis of desmosomal, fascia adherens and gap junction proteins at 4 weeks post injection in uninfected control, uninfected *PKP2* Hom and AAV-PKP2-treated *PKP2* Hom hearts. GAPDH served as the loading control. **e**, Quantification of protein expression in (**d**) normalized to GAPDH (controls, *n* = 5; Hom, *n* = 5; Hom-AAV-PKP2, *n* = 7 biologically independent animals). Data are presented as mean ± s.e.m. Two-way ANOVA with Tukey’s multiple comparison test. ****P < 0.0001, ***P < 0.001 (DSG2, *P* = 0.0008), *P < 0.05 (CX43, Ctrl versus Hom-AAV-PKP2, *P* = 0.0123; Hom versus Hom-AAV-PKP2, *P* = 0.0174). Experiments were repeated independently three times with similar results. **f**, Hematoxylin and eosin staining of cardiac sections from wild-type control and *PKP2* Hom mice treated with AAV-GFP or AAV-PKP2. Scale bars, 1 mm. **g**, Ratios of heart weight to body weight of wild-type control and *PKP2* Hom mice treated with AAV-GFP or AAV-PKP2 (controls, *n* = 6; Hom, *n* = 4; Hom-AAV-PKP2, *n* = 8 biologically independent animals). Data are presented as mean ± s.e.m. One-way ANOVA with Tukey’s multiple comparison test. ***P < 0.001 (*P* = 0.0008), **P < 0.01 (*P* = 0.0014). **h**, Masson’s Trichrome staining of cardiac sections from wild-type control and *PKP2* Hom mice treated with AAV-GFP or AAV-PKP2. Scale bars, 100 µm. **i**, RT–qPCR analysis of *Col1α1* levels (controls, *n* = 4; Hom, *n* = 4; Hom-AAV-PKP2, *n* = 6 biologically independent animals). Data are presented as mean ± s.e.m. One-way ANOVA with Tukey’s multiple comparison test. *P < 0.05 (*P* = 0.0316 for Ctrl versus Hom; *P* = 0.0429 for Hom versus Hom-AAV-PKP2). Source data

To determine whether restoring cardiac PKP2 protein levels can affect cardiac desmosomal protein disruption and disease in *PKP2* Hom mice in vivo, we designed an AAV strategy to express wild-type mouse *PKP2* (with carboxy-terminal tagged with FLAG; AAV-PKP2) in the heart using a cardiotropic AAV9 serotype and cardiac troponin T (cTnT) promoter (Fig. 5a). AAV9 tagged with green fluorescent protein (AAV-GFP) was used as a control. *PKP2* Hom mice were injected with AAV at 5 × 10^11^ genome copies per mouse on postnatal day 2 and subsequently analyzed at 4 weeks of age (Fig. 5b), a time point at which all ARVC disease features are present (Figs. 1 and 2). The virus dose was based on previous studies using neonatal AAV injection in a mouse model of catecholaminergic polymorphic ventricular tachycardia^32^. Immunofluorescence analysis showed FLAG staining localized at the cardiomyocyte cell–cell junction in AAV-PKP2-treated *PKP2* Hom hearts, as evidenced by colocalization with the desmosomal marker JUP within cardiomyocytes marked with alpha-actinin staining, compared to uninjected controls (Fig. 5c), demonstrating that exogenous PKP2 can localize to the cardiomyocyte cell–cell junction following AAV delivery. Western blot analyses revealed that neonatal administration of AAV-PKP2 was sufficient to restore PKP2 protein to endogenous levels in 4-week-old *PKP2* Hom hearts (Fig. 5d,e). PKP2 restoration was also sufficient to significantly increase levels of other desmosomal components (DSP, DSG2 and JUP), as well as of the integral gap junction component CX43 (Fig. 5d,e) in 4-week-old *PKP2* Hom hearts. The fascia adherens marker N-Cad remained unchanged at 4 weeks post AAV-PKP2 treatment, similar to untreated controls (Fig. 5d,e). These findings were in contrast to untreated *PKP2* Hom mice, which demonstrated significant desmosomal (PKP2, DSP, DSG2, JUP) dissolution and gap junction (CX43) protein reduction (Fig. 5d,e). These data further highlight the selective ability of *PKP2*, as a single desmosomal gene, to scaffold and reassemble cardiac cell–cell junction components at and beyond the cardiac desmosome.

To determine the effect of AAV-PKP2 on cardiac morphology and remodeling in *PKP2* Hom mice, we performed gross morphological and histological analyses. Hematoxylin and eosin staining of whole heart sections revealed that cardiac dimensions and cardiac muscle integrity were affected in *PKP2* Hom mice treated with AAV-GFP, which exhibited enlarged left and right ventricular chambers and extensive hematoxylin (nuclear) staining in the left ventricle free wall (Fig. 5f), suggestive of cardiomyocyte necrosis and calcification. By contrast, *PKP2* Hom mice treated with AAV-PKP2 were indistinguishable from wild-type controls, showing no evidence of disease (Fig. 5f). Ratios of heart weight to body weight revealed similar findings (Fig. 5g). However, *PKP2* Hom hearts treated with AAV-GFP displayed significantly higher ratios of heart weight to body weight compared to controls and AAV-PKP2-treated *PKP2* Hom mice (Fig. 5g). Masson’s Trichrome (collagen) stains revealed that cardiac sections from AAV-PKP2-treated *PKP2* Hom mice were indistinguishable from those from wild-type controls, suggesting the absence of fibrosis (Fig. 5h). By contrast, extensive fibrotic areas were present in both left and right ventricles of *PKP2* Hom mice treated with AAV-GFP (Fig. 5h). RT–qPCR for fibrotic gene expression (*Col1a1*) also revealed a prevention of fibrotic remodeling in *PKP2* Hom mice treated with AAV-PKP2, as their *Col1a1* levels were indistinguishable from those of controls (Fig. 5i). By contrast, *PKP2* Hom mice treated with AAV-GFP exhibited a significant induction of fibrotic gene expression (Fig. 5i). Together, these data suggest that AAV-PKP2 can prevent pathological cardiac remodeling and fibrosis in 4-week-old *PKP2* Hom mice.

**Fig. 6:**
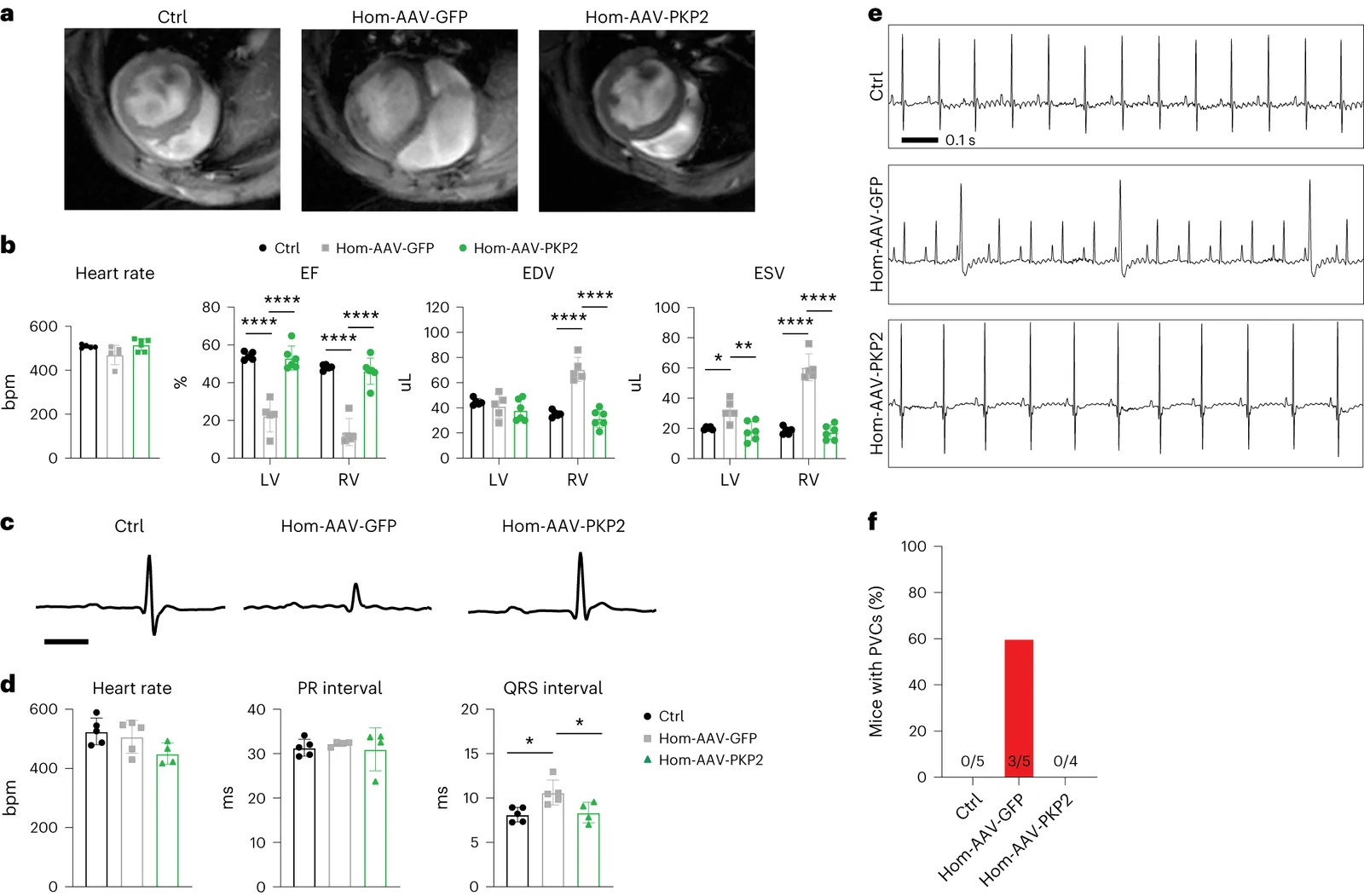
Early administration of AAV-PKP2 prevents cardiac mechanical and electrical pathological hallmarks of ARVC in *PKP2* Hom mice. **a**, Representative short-axis MRI from wild-type control mice, *PKP2* Hom mice treated with AAV-GFP and *PKP2* Hom mice treated with AAV-PKP2. **b**, Quantification of heart rate, ejection fraction, end-diastolic volume and end-systolic volume (controls, *n* = 5; Hom-AAV-GFP, *n* = 5; Hom-AAV-PKP2, *n* = 6 biologically independent animals). Data are presented as mean ± s.e.m. One-way ANOVA with Tukey’s multiple comparison test for heart rate measurement. Two-way ANOVA with Bonferroni’s multiple comparison test for additional measurements. Adjusted *P* values, ****P < 0.0001, **P < 0.01, *P < 0.05 (ESV, LV, Ctrl versus Hom-AAV-GFP, *P* = 0.0210; Hom-AAV-GFP versus Hom-AAV-PKP2, *P* = 0.0039). **c**, Representative composite surface ECG tracings averaged from four beats in wild-type control and *PKP2* Hom mice treated with AAV-GFP or AAV-PKP2 at 4 weeks of age. Scale bar, 10 ms. **d**, Quantification of heart rate, PR interval and QRS interval from composite surface ECG tracings (controls, *n* = 5; Hom-AAV-GFP, *n* = 5; Hom-AAV-PKP2, *n* = 4 biologically independent animals). Data are presented as mean ± s.e.m. One-way ANOVA with Tukey’s multiple comparison test. *P < 0.05 (Ctrl versus Hom-AAV-GFP, *P* = 0.0149; Hom-AAV-GFP versus Hom-AAV-PKP2, *P* = 0.0348). **e**, Representative ECG tracings through time from control and *PKP2* Hom mice treated with AAV-GFP or AAV-PKP2 at 4 weeks of age. **f**, Quantification of mice demonstrating PVCs (red arrows) (controls, *n* = 5; Hom-AAV-GFP, *n* = 5; Hom-AAV-PKP2, *n* = 4 biologically independent animals). Source data

To determine the functional and electrical effect of AAV-PKP2 in 4-week-old *PKP2* Hom mice in vivo, we performed cardiac MRI and surface ECG analyses. Representative short-axis MRI views highlighted the positive effect of AAV-PKP2 on cardiac dimensions at end-diastole, particularly in the right ventricle (Fig. 6a). Quantitative MRI analysis revealed no significant differences in heart rates between groups; however, cardiac function, assessed by ejection fraction, was significantly improved in both left and right ventricles following AAV-PKP2 treatment compared to AAV-GFP treatment (Fig. 6b). This was accompanied by significant decreases in right ventricular end-diastolic and end-systolic volumes as well as left ventricular end-systolic volumes in the AAV-PKP2-treated mice compared to controls (Fig. 6b). The analysis of composite surface ECG tracings (Fig. 6c) revealed a significantly widened QRS complex in AAV-GFP-treated *PKP2* Hom mice (Fig. 6d), which was significantly corrected to wild-type control values in *PKP2* Hom mice treated with AAV-PKP2 (Fig. 6d). Furthermore, no observed differences in heart rate or PR interval were found between groups (Fig. 6d), highlighting a specific effect of AAV-PKP2 in alleviating ventricular depolarization delay. Surface ECG analyses also revealed that 60% of AAV-GFP-treated *PKP2* Hom mice exhibited PVCs at 4 weeks of age (Fig. 6e,f), whereas 0% of wild-type control and 0% of *PKP2* Hom mice treated with AAV-PKP2 showed PVCs (Fig. 6e,f). Together, these data demonstrate that AAV-PKP2 can alleviate the severe cardiac electrical and mechanical deficits found in *PKP2* Hom mice.

### Early PKP2 restoration affords long-term ARVC protection

**Fig. 7:**
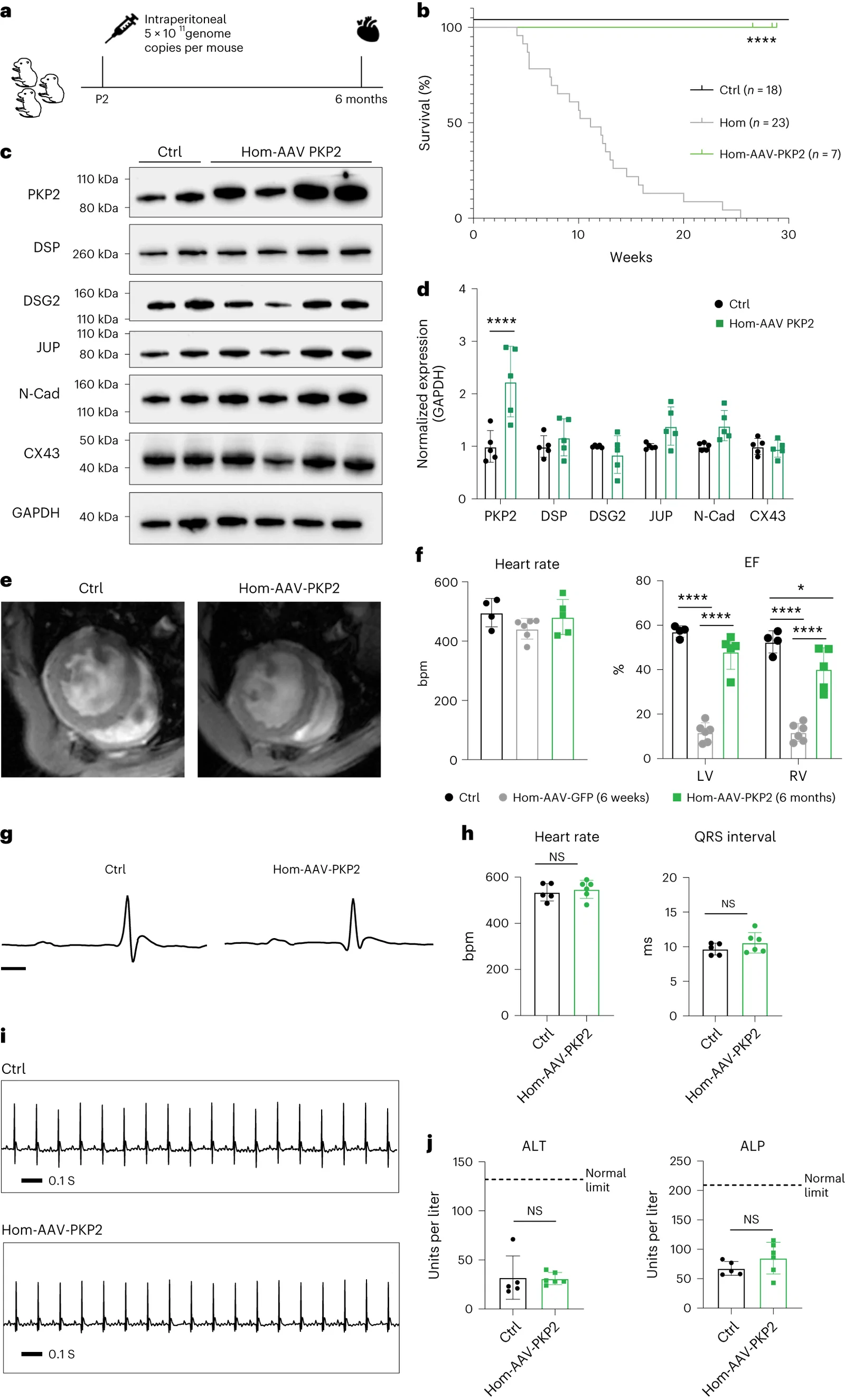
Early administration of AAV-PKP2 affords long-term cardiac desmosomal protection, function and survival in 6-month-old *PKP2* Hom mice. **a**, Schemata for early injection of AAV-GFP or AAV-PKP2 to *PKP2* Hom mice at postnatal day 2 and post analysis at 6 months. **b**, Kaplan–Meier survival analysis (log-rank test) of mice (controls, *n* = 18; Hom, *n* = 23; Hom-AAV-PKP2, *n* = 7 biologically independent animals). Ctrl and *PKP2* Hom data in Fig. 7 are also presented in Fig. 1. ****P < 0.0001 (*Χ*^2^ 58.07, d.f. 2). **c**, Western blot analysis of PKP2 and cell–cell junctional proteins (DSP, DSG2, JUP, N-Cad and CX43) in mouse hearts. GAPDH was used as a loading control. **d**, Quantification of protein expression shown in **c** normalized to GAPDH. Data are presented as mean ± s.e.m., *n* = 5 biologically independent animals. Two-way ANOVA with Sidak’s multiple comparison test. ****Adjusted *P* < 0.0001. **e**, Representative short-axis cardiac MRI views. **f**, Quantification of heart rate and ejection fraction in mice using cardiac MRI (controls, *n* = 4; Hom-AAV-GFP, *n* = 6; Hom-AAV-PKP2, *n* = 5 biologically independent animals). Data are presented as mean ± s.e.m. One-way ANOVA for heart rate comparison. Two-way ANOVA with Bonferroni’s multiple comparison test for other comparisons. Adjusted *P* values, ****P < 0.0001, *P < 0.05 (*P* = 0.0358). Historical data for Hom-AAV-GFP are from 6 weeks as no *PKP2* Hom mouse survived to 6 months of age. **g**, Representative composite surface ECG tracings averaged from four beats in untreated wild-type control and *PKP2* Hom mice treated with AAV-PKP2. Scale bar, 10 ms. **h**, Quantification of heart rate and QRS intervals from composite surface ECG tracings (controls, *n* = 5; Hom-AAV-PKP2, *n* = 6 biologically independent animals). Data are presented as mean ± s.e.m. Two-tailed unpaired *t*-test. NS, not significant. **i**, Representative ECG tracings from untreated control and *PKP2* Hom mice treated with AAV-PKP2. **j**, Blood serum analysis for ALT and ALP liver enzyme levels in untreated wild-type control and *PKP2* Hom mice treated with AAV-PKP2 (controls, *n* = 5; Hom-AAV-PKP2, *n* = 6 biologically independent animals). Normal enzyme limits are indicated with dotted lines. Data are presented as mean ± s.e.m. Two-tailed unpaired *t*-test. Source data

**Extended Data Fig. 6:**
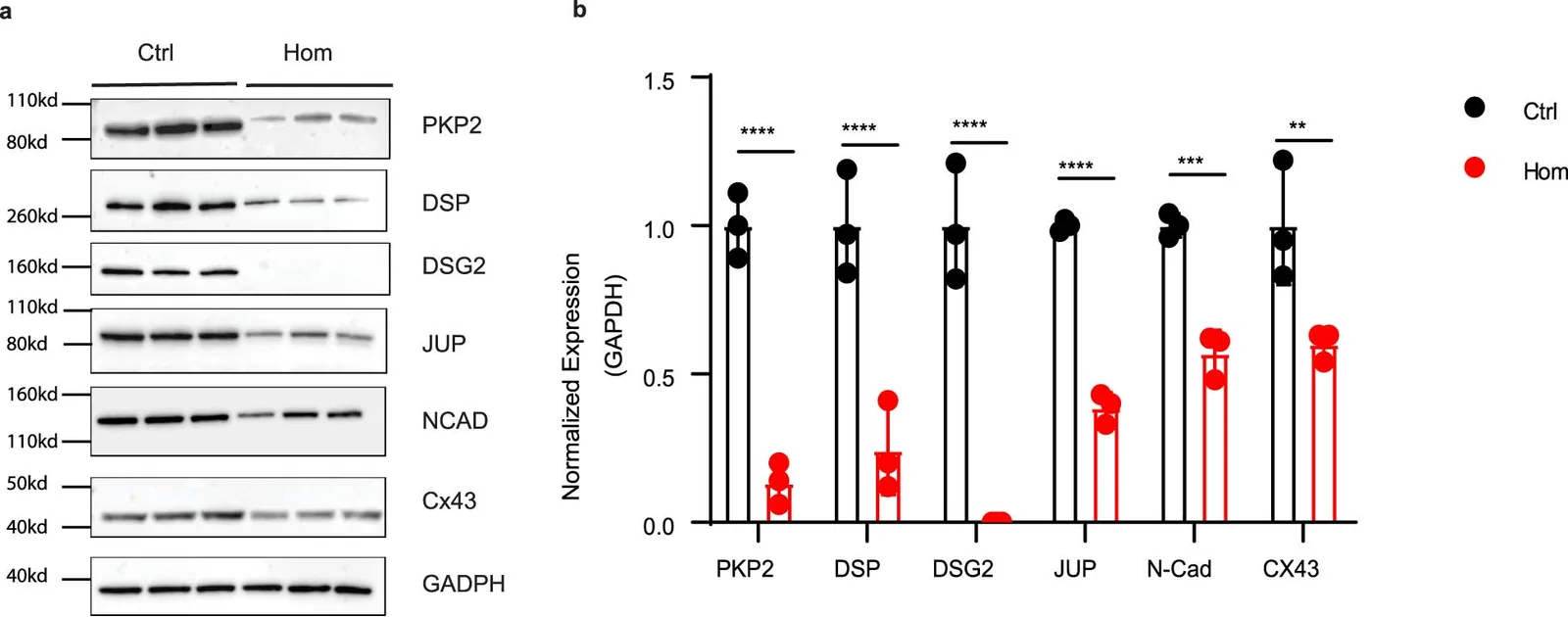
Late-stage dissolution of the cardiac cell-cell junction in PKP2 Hom hearts. **(a)** Western blot analysis of desmosomal, fascia adherens, and gap junction proteins at eight weeks of age in Ctrl and PKP2 Hom hearts. GAPDH serves as the loading control. **(b)** Quantification of protein expression in **(a)** normalized to GAPDH (n = 3 biologically independent animals per group). Data are presented as mean ± S.E.M. Two-way ANOVA with Tukey’s multiple comparison test. ****, p < 0.0001, ***, p < 0.001 (p = 0.0008), **, p < 0.01 (p = 0.0019). Source data

To determine the long-term effect of AAV-PKP2 treatment in *PKP2* Hom mice in vivo, we assessed cardiac cell–cell junction protein homeostasis, function and survival in mice up to 6 months of age (Fig. 7a). Kaplan–Meier survival analysis demonstrated 100% survival of AAV-PKP2-treated *PKP2* Hom mice at 6 months of age, which was indistinguishable from survival in wild-type littermate control mice (Fig. 7b). This finding is in stark contrast to results in untreated *PKP2* Hom mice, which exhibited a median survival of 11 weeks, with 0% survival at 6 months of age (Fig. 7b). Given that no untreated or AAV-GFP-treated *PKP2* Hom mouse survived to 6 months of age, data from AAV-PKP2-treated *PKP2* Hom mice were compared to data from wild-type littermate controls or to historical data from untreated or AAV-GFP-treated *PKP2* Hom mice at earlier disease stages as a reference. Western blot analysis revealed that AAV-PKP2 treatment prevented dissolution of the cardiac cell–cell junction in *PKP2* Hom mice (Fig. 7c,d), which was apparent as early as postnatal day 1–2 in untreated *PKP2* Hom mice (Extended Data Fig. 5). We found that PKP2 protein levels persisted in hearts from AAV-PKP2-treated *PKP2* Hom mice at 6 months of age compared to hearts from littermate controls (Fig. 7c,d). Persistent PKP2 expression prevented the loss of desmosomal (DSP, DSG2 and JUP), fascia adherens (N-Cad) and gap junction (CX43) proteins in 6-month-old *PKP2* Hom hearts treated with AAV-PKP2, as they were indistinguishable from littermate control hearts (Fig. 7c,d). By contrast, untreated *PKP2* Hom mice displayed a complete dissolution of the cardiac cell–cell junction, as shown by the significant reduction in desmosomal, gap junction and fascia adherens junction protein levels at 8 weeks of age compared to those of wild-type littermate control hearts (Extended Data Fig. 6). Representative short-axis MRI views revealed similar cardiac morphology between wild-type littermate control and AAV-PKP2-treated *PKP2* Hom mice (Fig. 7e). Quantification of cardiac function via ejection fraction highlighted no significant differences in left ventricular function between 6-month-old wild-type littermate controls and AAV-PKP2-treated *PKP2* Hom mice, with AAV-PKP2 treatment eliciting near-wild-type control levels in terms of right ventricular function in *PKP2* Hom mice (Fig. 7f). This finding is in marked contrast to that obtained in AAV-GFP-treated *PKP2* Hom mice, which showed a significant reduction in both left and right ejection fraction at 6 weeks of age (Fig. 7f), highlighting that AAV-PKP2 slows rapid cardiac dilatation and deterioration of cardiac function in *PKP2* Hom mice. An assessment of electrical function via composite surface ECG tracing analysis revealed that 6-month-old AAV-PKP2-treated *PKP2* Hom mice exhibited similar heart rates and QRS intervals to wild-type littermate controls, including the absence of PVCs (Fig. 7g,h). These data suggest that early postnatal administration of AAV-PKP2 prevents the electrical deficits, including ventricular depolarization delay and cardiac arrhythmias, in *PKP2* Hom mice in the long term (6 months). Given that the liver represents a major off-target site with AAV treatment, we assessed circulating levels of liver enzymes alkaline phosphatase (ALP) and alanine aminotransferase (ALT) as a measure of liver function. We observed no significant differences in the levels of either enzyme between wild-type littermate control and AAV-PKP2-treated *PKP2* Hom mice at 6 months of age (Fig. 7i), suggesting that liver function is not affected by long-term *PKP2* gene therapy. These data highlight that AAV-PKP2 can drive persistent PKP2 protein expression, which elicits long-term desmosomal protection to prevent severe electrical and mechanical cardiac dysfunction as well as premature lethality in *PKP2* Hom mice.

### Late PKP2 restoration rescues ARVC deficits in mice

**Fig. 8:**
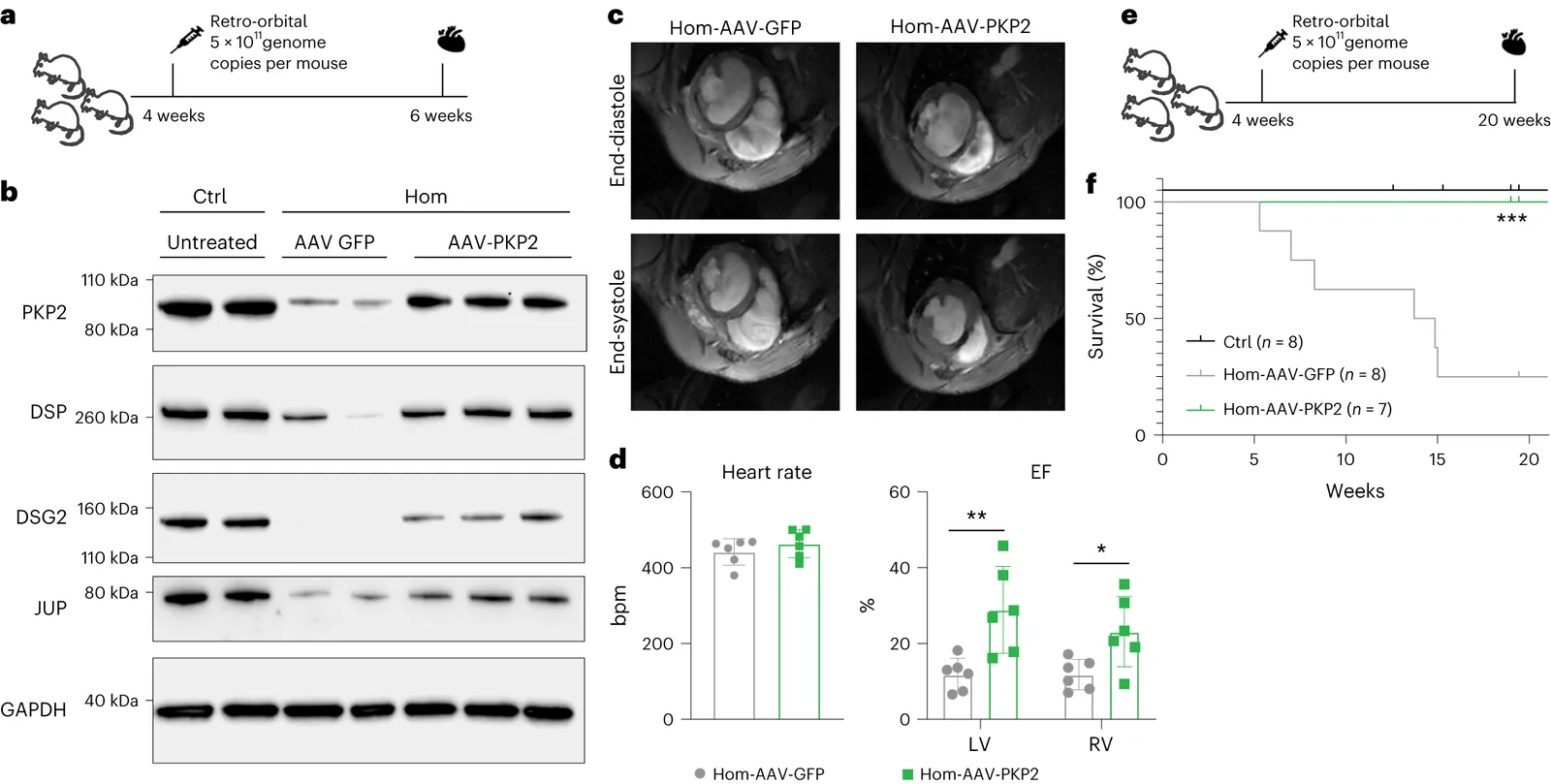
Late-stage administration of AAV PKP2 has an immediate impact on cardiac desmosomal deficits and function to promote late term survival in 5 month old PKP2 Hom mice. **a**, Schemata for late injection of AAV-GFP or AAV-PKP2 to *PKP2* Hom mice at 4 weeks and post analysis at 6 weeks. gc, genom copies. **b**, Western blot analysis of PKP2 and desmosomal cell–cell junctional proteins (DSP, DSG2 and JUP) in mouse hearts. GAPDH was used as a loading control. Experiments were repeated independently three times with similar results. **c**, Representative short-axis MRI views from mice. **d**, Quantification of heart rate and ejection fraction in mice using cardiac MRI (*n* = 6 biologically independent animals). Data are presented as mean ± s.e.m. Two-way ANOVA with Sidak’s multiple comparison test. **P < 0.01 (*P* = 0.0026), *P < 0.05 (*P* = 0.0450). **e**, Schemata for late injection of AAV-GFP or AAV-PKP2 to *PKP2* Hom mice at 4 weeks and post analysis at 20 weeks. gc, genom copies. **f**, Kaplan–Meier survival analysis (log-rank test) of mice (controls, *n* = 8; Hom-AAV-GFP, *n* = 8; Hom-AAV-PKP2, *n* = 7 biologically independent animals. ***adjusted *P* < 0.001 (*Χ*^2^ 16.59, d.f. 2, *P* = 0.0003). Source data

**Extended Data Fig. 7:**
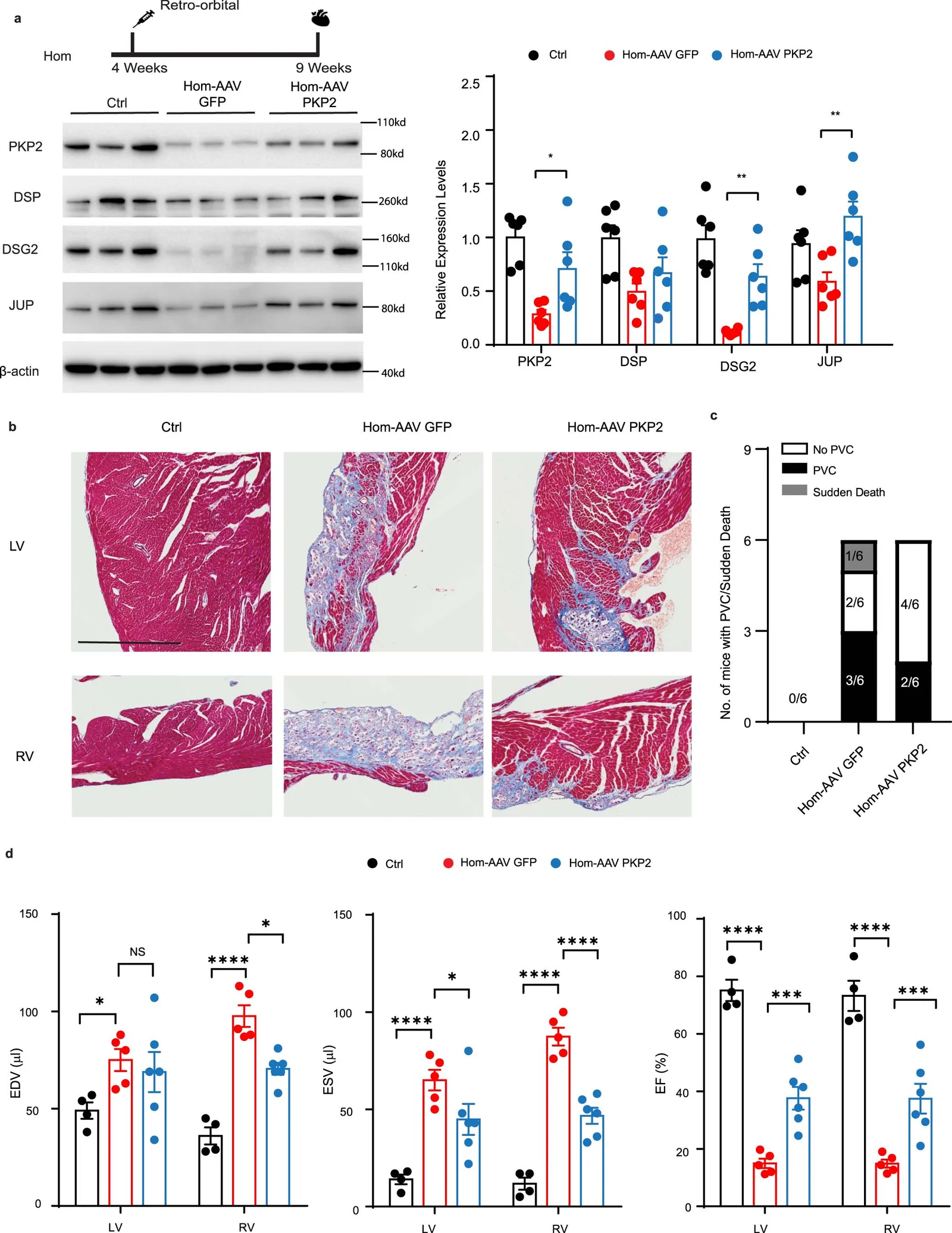
Longitudinal assessment of late-stage administration of AAV PKP2 reveals continued effects on alleviating cardiac desmosomal deficits in addition to reduction in cardiac histopathology (fibrosis), arrhythmias and dysfunction at five weeks post-AAV administration. (**a**) Schemata for late injection of AAV GFP or AAV PKP2 to PKP2 Hom mice at 4 weeks and post-analysis at 9 weeks (top). Western blot analysis of PKP2 and desmosomal proteins (DSP, DSG2 and JUP) in mouse hearts. β-actin used as a loading control. Data are presented as mean ± S.E.M. n = 6 biologically independent animals per group, Two-way ANOVA with Tukey’s multiple comparison test. *, p < 0.05 (PKP2, p = 0.0410), **, p < 0.01 (DSG2, p = 0.0099; JUP, p = 0.0020). **(b)** Representative Masson’s Trichrome stains from left (LV) and right ventricular (RV) sections from 9-week-old Ctrl, PKP2 Hom-AAV GFP (five weeks post-late stage administration) and PKP2 Hom-AAV PKP2 (five weeks post-late stage administration) mice. Scale bar, 2 mm. Experiments were repeated independently three times with similar results. (**c**) Quantification of mice demonstrating PVCs and sudden death at 9 weeks of age in Ctrl, PKP2 Hom-AAV GFP (five weeks post-late stage administration) and PKP2 Hom-AAV PKP2 (five weeks post-late stage administration) mice. **(d)** Quantification of end-diastolic volumes (EDV), end-systolic volumes (ESV) and percentage of ejection fraction (EF %) in 9 week old Ctrl, PKP2 Hom-AAV GFP (five weeks post-late stage administration) and Hom-AAV PKP2 (five weeks post-late stage administration) mice using cardiac MRI. Data are presented as mean ± S.E.M, n = 4 for Ctrl, n = 5 for Hom-AAV GFP group and n = 6 for Hom-AAV PKP2 biologically independent animals. Two-way ANOVA with Tukey’s multiple comparison test. ****, adjusted p < 0.0001. ***, p < 0.001 (EF, LV, p = 0.0008; RV, p = 0.0008). *, p < 0.05 (EDV, LV, p = 0.0369; RV, p = 0.0148; ESV, LV, p = 0.0352), ns, not significant. Source data

**Extended Data Fig. 8:**
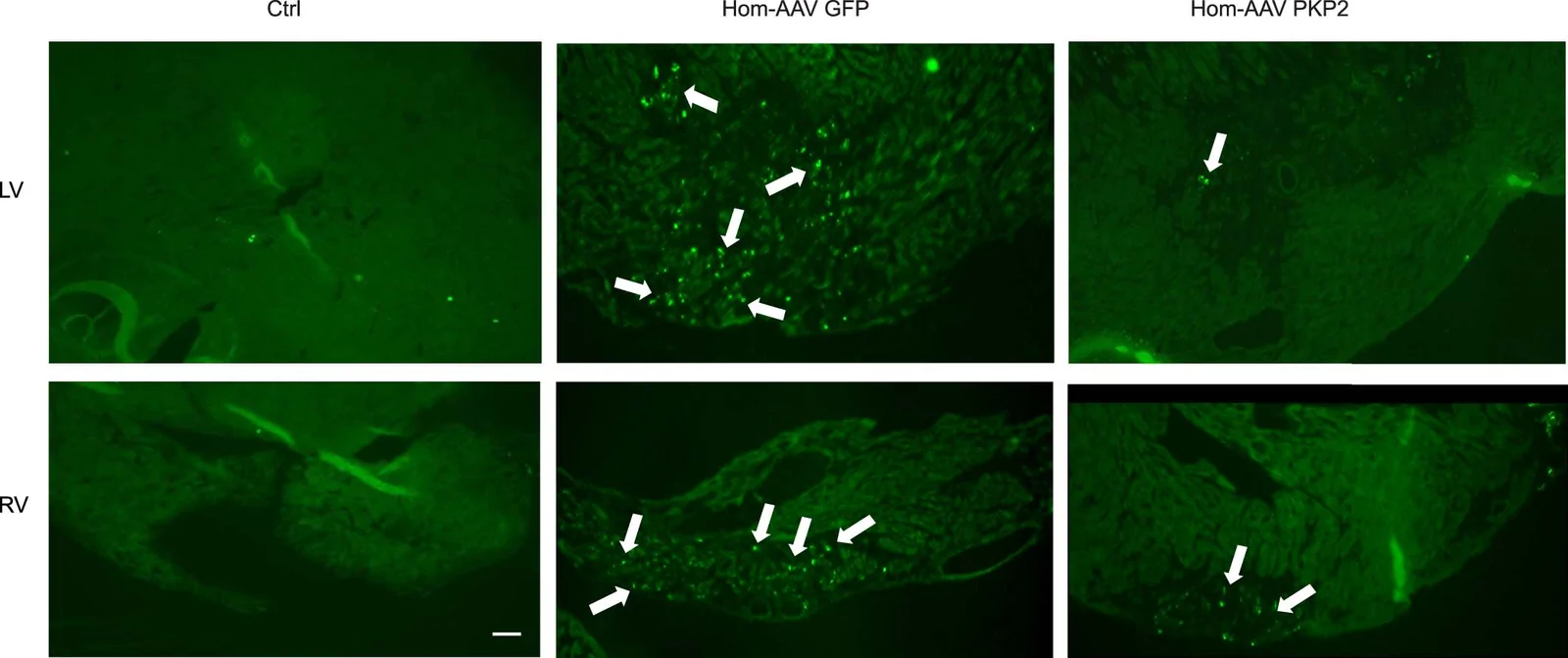
PKP2 Hom mice display fat deposition that can be alleviated with late-stage administration of AAV PKP2. Immunofluorescence microscopy analysis of perilipin (adipocyte marker) expression and localization in representative cardiac sections from 9 week old WT as well as PKP2 Hom mice at five weeks post-late stage administration of AAV GFP or AAV PKP2. White arrows denote increased number of positive perilipin stains in PKP2 Hom mouse hearts treated with AAV GFP and significant reduction in perilipin positive stained regions in PKP2 Hom mouse hearts treated with AAV PKP2. Scale bar = 30 μm. Experiments were repeated independently three times with similar results.

**Extended Data Fig. 9:**
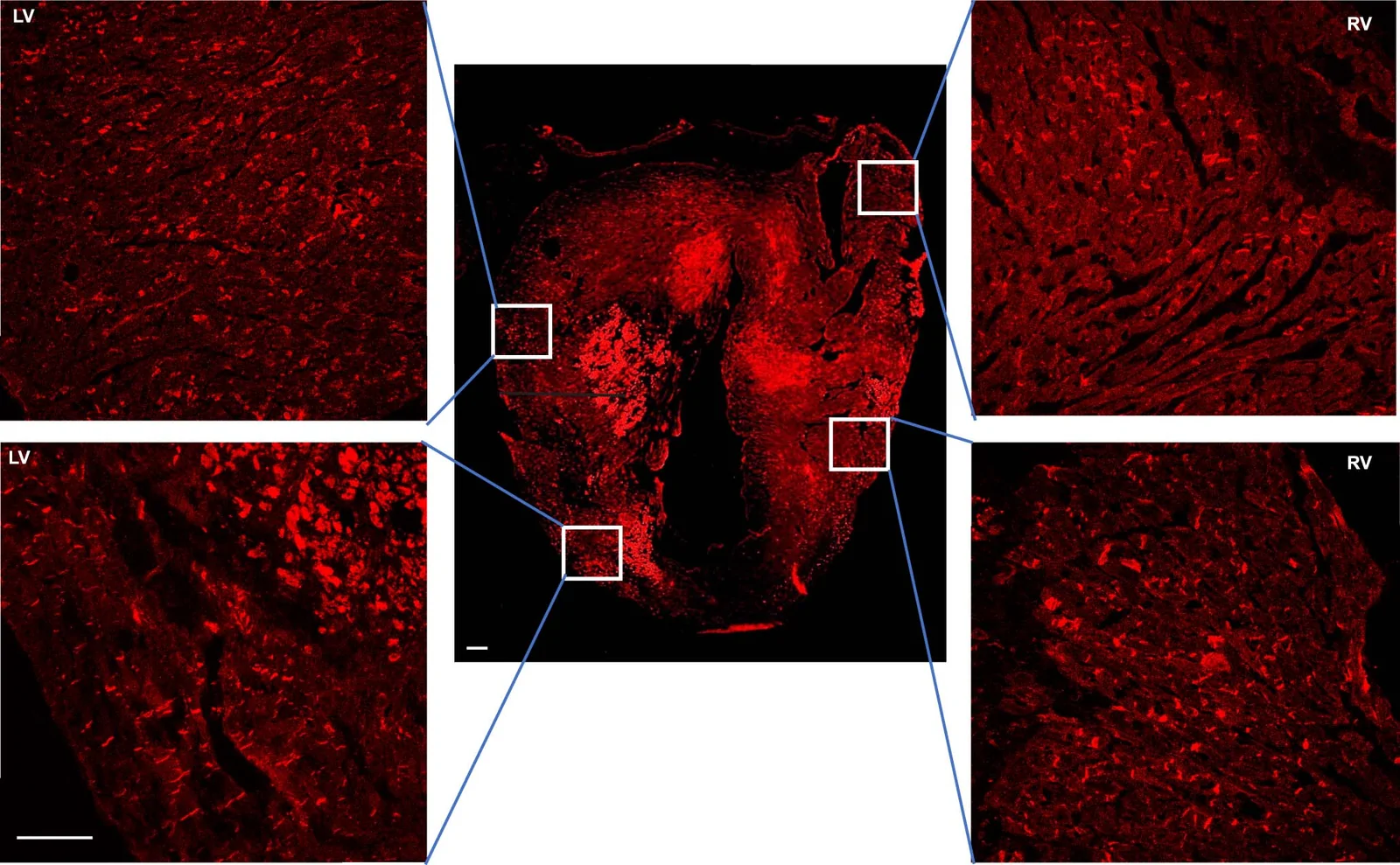
PKP2 Hom mice display AAV PKP2-FLAG expression and distribution in a majority of cardiomyocytes throughout the right and left ventricles. Immunofluorescence microscopy analysis of FLAG expression and localization in representative cardiac sections from 9 week old PKP2 Hom mice at five weeks post-late stage administration of AAV PKP2. Scale bar = 500 μm. Two insets on the left represent high magnification views of cardiomyocytes from left ventricle. Two insets on the right represent high magnification views of cardiomyocytes from right ventricle. Please note the localization of FLAG signal to the cell-cell junction in the inset panels. Inset scale bar, 100 μm. Experiments were repeated independently three times with similar results.

To evaluate the effect of *PKP2* gene therapy in the setting of existing ARVC, *PKP2* Hom mice were injected with AAV at 5 × 10^11^ genome copies per mouse at 4 weeks of age (Fig. 8a), a time point at which all gross ARVC disease features are present (Figs. 1 and 2). Mice were euthanized 2 weeks (Fig. 8a) and 5 weeks (Extended Data Fig. 7) post AAV administration to assess early and longitudinally late ARVC molecular and functional outcomes. As early as 2 weeks post AAV injection, AAV-PKP2 treatment was sufficient to increase PKP2 levels and significantly improve desmosomal protein (DSP, DSG2 and JUP) levels in *PKP2* Hom mice compared to those in AAV-GFP-treated *PKP2* Hom mice (Fig. 8b). In addition, representative cardiac short-axis MRI views at both end-diastole and end-systole showed an improvement in cardiac morphology in AAV-PKP2-treated *PKP2* Hom mice at 6 weeks of age compared to littermate AAV-GFP-treated *PKP2* Hom mice (Fig. 8c). Quantitative MRI analysis revealed no significant differences in heart rates between mice, but it showed a significant improvement in left and right ventricular ejection fraction in AAV-PKP2-treated *PKP2* Hom mice compared to AAV-GFP-treated *PKP2* Hom mice (Fig. 8d). We also found that as late as 5 weeks post AAV injection, AAV-PKP2 treatment retained the ability to alleviate desmosomal protein loss, resulting in longitudinal effects on alleviating cardiac fibrofatty deposition and arrhythmias, as well as further improving cardiac right and left mechanical function in adult *PKP2* Hom mice compared to AAV-GFP-treated controls (Extended Data Figs. 7 and 8). Immunofluorescence microscopy also revealed Nav1.5 protein relocalization to the cell–cell junction and membrane in AAV-PKP2-treated *PKP2* Hom mice compared to AAV-GFP-treated controls (Extended Data Fig. 2). Furthermore, NF-κB protein expression and specific sets of cardiac inflammatory cytokines were also downregulated in hearts and serum of AAV-PKP2-treated *PKP2* Hom mice, respectively, compared to AAV-GFP-treated controls (Extended Data Figs. 3 and 4). Survival analysis revealed 100% survival of AAV-PKP2-treated *PKP2* Hom mice at 20 weeks of age compared to 20% survival of AAV-GFP-treated *PKP2* Hom mice at the same age (Fig. 8e,f). Immunofluorescence microscopy of FLAG expression showed that this one-time late-stage administration of AAV-PKP2 (which was tagged with FLAG) at 5 weeks post AAV administration was sufficient to infect a majority of cardiomyocytes in the left and right ventricles of *PKP2* Hom mice (Extended Data Fig. 9), concurrent with improvements in cardiac desmosomal biology, histopathology, rhythm and physiology in *PKP2* Hom mice at these late stages. These results highlight the restorative actions and durability of AAV-PKP2 to rescue multiple parameters associated with ARVC deficits, which resulted in prolongation of lifespan of *PKP2* Hom mice, even when administered in advanced stages of ARVC.

## Discussion

In this study, we generate a *PKP2* genetic knock-in mouse model to demonstrate the sufficiency of a prevalent *PKP2* RNA splicing mutation (human *PKP2* IVS10-1G>C) to phenocopy key human ARVC features in mouse, including sudden death, ventricular arrhythmias (which include ventricular depolarization abnormalities serving as a primer for life-threatening arrhythmias, as well as electrophysiological alterations in sodium channel function and Nav1.5 protein localization), biventricular dysfunction, inflammation, desmosomal ultrastructural deficits and fibrofatty replacement of myocardium. Although we acknowledge that more research is needed to fully understand the extent to which the observed changes in *PKP2* Hom mice are present in humans, we believe our study represents an important step in unraveling complex mechanisms underlying *PKP2*-related ARVC, especially related to splicing mutations, providing a foundation for future investigations in both animal models and clinical settings. Furthermore, we show that early- and late-stage administration of *PKP2* gene therapy can be used as a means to circumvent and rescue ARVC development and mortality in this genetic *PKP2* model that harbors patient genetics. Our studies show that the *PKP2* IVS10-1G>C splice site mutation has direct consequences on *PKP2* RNA quality (that is, appearance of a larger, mutant PKP2) and levels (that is, reduced levels), suggestive of partial intron retention as a mechanism. Interestingly, this partial intron retention remains in frame and is successfully translated to a higher molecular weight mutant PKP2 protein at reduced levels of endogenous PKP2. Previous work suggests that intron retention mechanisms decrease transcript levels through a frameshift and the introduction of a premature termination codon (PTC)^18^. PTCs are known to trigger rapid mRNA degradation through a quality control mechanism termed ‘nonsense-mediated decay’, ultimately reducing mutant transcript levels to prevent translation of a potential toxic or gain-of-function protein^17,33^. Our data imply that mutant *PKP2* transcripts in *PKP2* Hom hearts are PTC-free, which suggests the possibility that non-PTC-associated mechanisms decrease *PKP2* transcript levels due to altered splicing kinetics from a weaker alternative intronic splice acceptor site. Future studies will focus on understanding the precise mechanisms by which altered *PKP2* splicing drives *PKP2* RNA consequences in *PKP2* Hom mice.

The unique PKP2 RNA and protein consequences of the *PKP2* IVS10-1G>C splice site mutation confer postnatal viability and desmosomal deficits leading to ARVC disease features in *PKP2* Hom mice. The presence of mutant PKP2 protein at reduced levels at the cardiac cell–cell junction in *PKP2* Hom hearts in vivo, and the findings of in vitro expression studies using mutant PKP2 in *PKP2* Hom neonatal cardiomyocytes reveal that mutant PKP2 is retained and functional at the cell–cell junction and capable of restoring desmosomal protein levels. This probably accounts for the viability of *PKP2* Hom mice during embryonic development. This is in line with studies performed in global *PKP2* knockout mice, which demonstrated an embryonic lethal phenotype^34^, suggesting that the global loss of endogenous PKP2 offers no survival advantage to mice. Our data may also help to explain survival in transgenic mice expressing truncated mutant PKP2 (S329X) and AAV-mediated expression of truncated mutant PKP2 (R735X)^35,36^, as these models retained the presence of endogenous PKP2, which probably conferred a survival advantage. Another example of a desmosomal variant resulting in mutant byproducts that confer an advantage in terms of survival and disease-free status was shown in a mouse model harboring a JUP variant associated with Naxos disease, which is an autosomal recessive form of ARVC^37,38^. Characterization of *JUP*-mutant mice revealed that they expressed a truncated mutant JUP protein at significantly reduced levels compared to controls^38^. More importantly, generation of a model that increased levels of the truncated JUP protein to endogenous levels was sufficient to prevent disease development, further suggesting that the mutant JUP protein could function equivalent to the endogenous JUP protein in the heart^38^. Our data suggest that the molecular consequences of the *PKP2* IVS10-1G>C splice acceptor site mutation on PKP2 protein levels during early cardiac development may confer more pronounced ARVC-related cardiac phenotypes (that may not be fully compensated by the mutant PKP2 protein), as we observed pronounced baseline arrhythmias and fatty replacement in conjunction with cardiac mechanical and pathological dysfunction in the *PKP2* Hom model compared to other models that ablate or express mutations in *PKP2* in adult heart settings^35,36,39^. Importantly, our data add to the field and provide direct evidence that desmosomal protein disruption associated with the *PKP2* splice site mutation is driven by a loss of function (dose or amount of PKP2) mechanism over time and the progressive nature of ARVC. This is in line with a growing number of human studies that suggest haploinsufficiency as a common mechanism that may underlie ARVC associated with various *PKP2* variants, including insertion or deletion, nonsense and missense subtypes^12,13^. As human patients with ARVC are heterozygous for the *PKP2* IVS10-1G>C mutation, our data also contribute to the growing evidence that a two-hit hypothesis (additional pathogenic stimulus) is central to elicit rapid ARVC pathogenesis by way of an additional genetic (a second desmosomal mutation or homozygous mutation) or physiological (for example, exercise stress) hit, especially in mouse settings^8,24,40^. This is further exemplified in *PKP2* Het mutant mice, which exhibited a latent phenotype on survival and primarily exhibited arrhythmias in the absence of cardiomyopathy when assessed over the same and longer timeframes as *PKP2* Hom mice. These electrophysiological abnormalities may probably account for premature lethality and sudden cardiac death in *PKP2* Het mice at later ages. Furthermore, our previous studies also highlight the requirement for two human desmosomal mutations—this *PKP2* RNA splice site (IVS10-1G>C) mutation and a *DSP* mutation; and thus, a compound heterozygous mutation—in order to elicit early and severe ARVC in a heterozygous setting in mouse in vivo^28^. Regardless, *PKP2* Het mutant mice will provide a unique model to exacerbate and tease out the arrhythmogenic substrate and mechanisms underlying ARVC in the absence of cardiomyopathy in the future, which is also reminiscent of the early concealed phase of ARVC.

ARVC is an incurable genetic heart disease with significant mortality through sudden cardiac death and progressive heart failure. Studies in *PKP2* Hom mice most importantly show broad applicability of therapeutic approaches to restore PKP2 RNA and protein levels (through *PKP2* gene therapy) for *PKP2* ARVC populations, including splice site mutations. In vivo-based AAV-PKP2 strategies were sufficient to restore PKP2 in the early stages of ARVC development in *PKP2* Hom mice, which was sufficient to prevent not only the striking cardiac desmosomal dissolution but also gap junction deficits that together drive the long-term electrical (arrhythmias) and structural (cardiac pathology and biventricular dysfunction) cardiac deficits in ARVC in *PKP2* Hom hearts. This was further exemplified in the longitudinal late-stage administration studies, which showed that AAV-PKP2 could revert multiple histopathological cardiac abnormalities (reduction in cardiac fibrosis, fat deposition, inflammation and cytokine production) and physiological cardiac abnormalities (improvements in left and right ventricular dimensions and function, alleviation of cardiac arrhythmias) in *PKP2* Hom mice. Interestingly, longitudinal studies at 5 weeks post AAV-PKP2 administration showed further improvements in mechanical function in *PKP2* Hom mice compared to 2 weeks post AAV-PKP2 administration, highlighting a normalization of mechanical dysfunction as a function of time. The alleviation of cardiac arrhythmias was also supported by Nav1.5 protein relocalization to cell–cell junctions in *PKP2* Hom hearts; however, future studies focused on evaluating the biophysical properties of the sodium channel will be required to fully determine its importance in the rescue of the electrophysiological abnormalities associated with *PKP2*-related disease. Regardless, cardiac desmosomal restoration and improvement in mechanical function are critical factors in preventing arrhythmias and maintaining cardiac health, and therefore can contribute to circumventing abnormalities that could lead to arrhythmias.

These data further highlight the ability of PKP2 to reconstitute the desmosome postnatally and function as a complex scaffold. PKP2 has a selective affinity for desmosomal proteins and is known to interact with the cytoplasmic tails of the transmembrane desmosomal cadherins (DSG2 and DSC2), as well as the plakin protein DSP within the desmosomal complex^9,41^. JUP, similar to PKP2, is an armadillo protein thought to also be involved in linking the desmosomal cadherins and DSP^9,41^. The partial intron retention within the mutant PKP2 protein does not contain any critical structural domains and is located towards the C terminus of PKP2. The amino-terminal head domain is known to be essential for the interaction of PKP2 with desmosomal components^42,43^, therefore it is unlikely that the mutant PKP2 protein directly disrupts desmosomal interactions but can certainly lessen them, and therefore, loss of interactions are probably due to the loss of PKP2 protein dose. This concept is further exemplified in global PKP2 knockout mice^34^; PKP2 protein loss in this setting could recapitulate the key desmosomal protein deficits found in neonatal and adult *PKP2* Hom hearts. Thus, our findings provide evidence for the importance of PKP2 quantity in maintaining normal levels of cell–cell junction components (mutant PKP2 may preserve interaction to a certain degree but may be destabilizing postnatally as lower levels of mutant protein are expressed), supporting the concept of a critical threshold for PKP2-mediated regulation of cell–cell junction protein expression. Future studies focused on dissecting the molecular interactions and regulatory functions of PKP2 in controlling cell–cell junction component expression and determining whether the role of PKP2 is solely structural or extends to the control of protein expression will provide deeper understanding of the complex role of PKP2 in the pathogenesis of ARVC. The most striking effect of *PKP2* gene therapy in *PKP2* Hom mice is ensuring 100% survival when administered in early and late stages of the disease. Although the mechanisms remain to be investigated in detail, one possible mechanism could relate to its interactions with the desmosomal resident protein CSN6, which is thought to interact with PKP2 and DSP and restrict desmosomal protein degradation, as well as circumvent ARVC and premature death in mice^28^, and thus improve desmosomal protein half-life over time. Therefore, future studies should focus on uncovering whether CSN6 functions are integral to the circumvention of ARVC deficits and premature lethality associated with *PKP2* gene therapy in *PKP2* Hom mice. Given that the desmosome represents a primary molecular trigger for the pathogenesis of ARVC, the ability to reassemble this complex is critical for ARVC-targeted therapies. This approach goes beyond current strategies that focus on symptomatic relief of electrical deficits (that is, arrhythmias), by focusing on alleviating the structural and functional deficits as well as premature death that underlie ARVC.

In summary, AAV-PKP2 therapy represents an effective approach for both preventing and stabilizing ARVC disease progression in *PKP2* Hom mice. The use of a cardiotropic AAV serotype and cardiac-specific cTnT promoter enable durable PKP2 expression in the heart with no adverse effects on the liver. Further evaluation of additional tissue types is required before ultimate application of this approach in clinical settings. Optimization of dose (and potential of AAV antibodies) and capsid to determine maximum phenotypic improvement within a range that could be safely administered to human patients will be required to ensure the safety, efficacy and feasibility of gene therapy strategies for successful translation to patient populations with ARVC.

## Methods

All research and animal procedures were in full compliance with the ethical guidelines of the Institutional Animal Care and Use Committee of the University of California San Diego, and carried out in accordance with the Guide for the Care and Use of Laboratory Animals of the National Institutes of Health (NIH).

### Generation of experimental animals

Mouse lines harboring heterozygous *PKP2* IVS10-1G>C mutation were previously generated using CRISPR–Cas9-mediated methods and used for this study^28,44^. The mutation template used to generate *PKP2* IVS10-1G>C Het mice is presented in Fig. 1. Genomic DNA was extracted from mouse tails, and genomic fragments at target sites were amplified by PCR and sequencing. Genotype-positive knock-in mice were backcrossed with C57BL/6J mice (The Jackson Laboratory) for at least three generations to minimize potential off-target effects. *PKP2* IVS10-1G>C Het mice were crossed to generate controls, *PKP2* IVS10-1G>C Het, and *PKP2* IVS10-1G>C Hom mutant mice. Both male and female mice were used for all studies. Environmental controls were set to maintain a temperature of 21–23 °C with a relative humidity of 45–55%. Temperature and humidity were monitored and a 12 h–12 h light–dark cycle was maintained, alternating at 6:00 and 18:00.

### Magnetic resonance imaging

In vivo cardiac MRI was performed on a 7T horizontal bore MR scanner (Bruker) as previously described^28^. In brief, a quadrature volume coil (Bruker) was used for radiofrequency signal transmission and a two-channel surface array coil (RAPID MRI) was used for reception of the RF signal. Cardiac CINE images were acquired with an IntraGate (Bruker) retrospective gated two-dimensional gradient echo pulse sequence (FLASH) with the following parameters: TE = 3.1 ms, TR = 5.6 ms, flip angle = 7°, 300–400 repetitions and 20 frames. A field of view of 2.0 × 1.5 cm and a data matrix of 256 × 192 were specified for a spatial resolution of 0.078 mm per pixel. Equatorial frames containing the largest and smallest chamber diameters were selected to define the end-diastolic and end-systolic times, respectively. For MRI image analyses, two-dimensional endocardial contours were manually segmented and slice volumes and/or ejection fractions calculated using freely available software (Segment CMR version 4.0 R12067, MEDVISO) for each heart at end-diastole and end-systole (both left and right ventricle). Volumes from continuous slices were summed to generate total chamber volumes at end-diastole and end-systole. Ejection fractions were averaged over all continuous slices.

### Surface and telemetry ECG

Surface ECG was performed as previously described^24^. In brief, mice were anesthetized with 5% isoflurane (Piramal, 6679401710) for 15 s and maintained at 1.5% isoflurane during the procedure. Needle electrodes (30-gauge) were inserted subcutaneously into the right forearm and left leg. Electrical activity was recorded for 5 min. For analysis, composite ECG tracings were generated for 100 consecutive beats and wave parts manually identified to generate heart rates, PR intervals and QRS intervals. For PVC analysis, ectopic beats were identified over the entire 5-min recording. For telemetry ECG recording, ECG transmitters (DSI) were subcutaneously inserted into the back of mice. After 5 days post surgery, ECGs were recorded for 3 days. The number of PVCs was analyzed using LabChart version 8.0.8 (ADINSTRUMENTS).

### Histological analysis

Mouse hearts were perfused in a relaxation buffer consisting of 300 mM KCl (Sigma-Aldrich, P9333) in PBS and fixed with 4% paraformaldehyde (ThermoFisher Scientific, J61899.AK). Fixed hearts were embedded in Tissue-Tek O.C.T. Compound (Sakura, 4583) or dehydrated and embedded in paraffin as previously described^24^. Sections of between 5 µm and 10 μm thickness were cut. Whole-heart (5 μm) paraffin sections were stained with hematoxylin and eosin (Sigma-Aldrich, HT1079-1SET) and Masson’s Trichrome (Sigma-Aldrich, HT15) stains according to the manufacturer’s instructions. Whole-heart (10 μm) cryosections were stained with Oil Red O (Sigma-Aldrich, O0625) according to the manufacturer’s instructions. Images were acquired with the Hamamatsu Nanozoomer 2.0 HT Slide Scanner.

### RNA analysis

Total RNA was isolated from hearts using TRIZOL (Invitrogen) according to the manufacturer’s instructions. The first-strand cDNA was generated using PrimeScript RT Reagent Kit with gDNA Eraser (Takara). RT–qPCR was performed using primer sequences (Extended Data Table 1) obtained from Integrated DNA Technologies and with KOD Extreme Hot Start DNA Polymerase (Sigma-Aldrich, 71975-3). RT–qPCR was performed on heart cDNA using primer sequences (Extended Data Table 1) with Power SYBR Green PCR master mix (Applied Biosystems, 43-091-55) and using a Bio-Rad Mastercycler. All values were normalized to GAPDH mRNA levels as indicated. PCR products were sequenced (Eton Biosciences).

### Protein analysis

Total protein extracts were isolated from cardiomyocytes and ventricles as previously described^24^. Immunodetection of desmoplakin (mouse, 1:1,000, Bio-Rad, 2722-504), desmoglein 2 (mouse, 1:1,000, Fitzgerald, 10R-D106a), plakophilin 2 C-terminal (mouse, 1:2,000, Fitzgerald, 10R-P130b), plakophilin 2 N-terminal (goat, 1:1,000, Lifespan Biosciences, LS-B9231), plakoglobin (goat, 1:1,000, Sigma-Aldrich, SAB2500802), N-cadherin (rabbit, 1:1,000, Abcam, ab76057), connexin 43 (rabbit, 1:8,000, Sigma-Aldrich, C6219), Nav1.5 (rabbit, 1:500, Alomone Labs, ASC-005), NF-κB (rabbit, 1:500, Santa Cruz Biotechnology, sc-372), β-actin (mouse, 1:2,000, Santa Cruz Biotechnology, sc-47778) and glyceraldehyde 3-phosphate dehydrogenase (mouse, 1:2,000, Santa Cruz Biotechnology, sc-32233) was done as previously described^24^. Briefly, secondary antibodies were used based on antibody species and included donkey anti-rabbit IgG (H+L) HRP (1:100, ThermoFisher Scientific, A16023), donkey anti-mouse IgG (H+L) HRP (1:100, ThermoFisher Scientific, A16011) and donkey anti-goat IgG (H+L) HRP (1:100, ThermoFisher Scientific, A15999) according to the manufacturer’s instructions.

### Immunofluorescence microscopy

Heart cryosections were fixed in 100% acetone (Fisher Scientific, A18-1) at −20 °C for 8 min and were blocked in 5% donkey serum (Sigma-Aldrich, D9663) or PBS (Gibco, 10010023) before incubation with antibodies. Sections were subsequently stained with primary antibodies against PKP2 (mouse, 1:100, Fitzgerald, 10R-P130b), desmoplakin (mouse, 1:100, Bio-Rad, 2722-504), plakoglobin (goat, 1:100, Sigma-Aldrich, SAB2500802), connexin 43 (rabbit, 1:100, Sigma-Aldrich, C6219), N-cadherin (rabbit, 1:100, Abcam, C6219), Nav1.5 (rabbit, 1:100, Alomone labs, ASC-005), FLAG (mouse, 1:100, Sigma-Aldrich, F3165), perilipin (rabbit, 1:100, Cell Signaling Technology, 3470S), sarcomere alpha actinin (mouse, Sigma-Aldrich, A7811), sarcomere alpha actinin (rabbit, abcam, ab68167) and secondary antibodies, such as donkey anti-mouse IgG (H+L) DyLight 488 (1:400, ThermoFisher Scientific, SA5-10166), donkey anti-mouse IgG (H+L) Alexa Fluor 555 (1:400, ThermoFisher Scientific, A31570), donkey anti-rabbit IgG (H+L) Alexa Fluor 488 (1:400, ThermoFischer Scientific, A21206), donkey anti-rabbit IgG (H+L) Alexa Fluor 568 (1:400, ThermoFisher Scientific, A10042) and donkey anti-goat IgG (H+L) Alexa Fluor 647 (1:400, ThermoFischer Scientific, A21447). Immunofluorescence images were acquired using a Leica SP8 confocal microscope.

### Neonatal cardiomyocyte isolation

Ventricular cardiomyocytes were isolated from neonatal (1–2 days old) mouse hearts using collagenase type II (Worthington Biochemical Corporation, LS004174) or trypsin (Sigma-Aldrich, T4799) digestion methods and plated on laminin (Gibco, 23-017-015) as previously described^24^. Cardiomyocytes were subsequently infected with adenoviruses expressing either wild-type PKP2 (MOI 1.0) or mutant PKP2 (MOI 1.0) for 24 h and subsequently maintained in media consisting of DMEM (Life Technologies, 11330-032), M199 (Corning, 10-060), 5% FBS (Gibco, 16000069), 10% horse serum (Gibco, 16050122) and 1% penicillin-streptomycin-glutamine (ThermoFisher Scientific, 10378016). For protein analyses, cardiomyocytes were collected at 5 days post infection.

### Adenovirus and adeno-associated virus vectors

Adenovirus vectors expressing either wild-type or mutant PKP2 were designed and synthesized containing a cytomegalovirus promoter, C-terminal PKP2 FLAG tag, and P2A-linked GFP (Vector Builder). Adenovirus was packaged and grown through the University of California San Diego viral vector core as previously described^24^. AAV vectors expressing wild-type PKP2 were designed and synthesized containing a cTnT promoter and C-terminal PKP2 FLAG tag (Vector Builder). In addition, AAV vector expressing GFP was designed and synthesized as a control. Viral particles were packaged into a cardiotropic AAV9 serotype and grown through the University of California San Diego viral vector core as previously described^45^.

### Adeno-associated virus injections

Early AAV injections were performed on postnatal day 2 using a 31-gauge needle and syringe (Monoject, 8881600800) to deliver 5 × 10^11^ viral particles per mouse in 50 µl of solution containing AAV9-GFP or AAV9-PKP2 via intraperitoneal injection. Late-stage AAV injections were performed at 4 weeks of age using a 31-gauge needle and syringe to deliver 5 × 10^11^ viral particles per mouse in 50 µl of solution containing AAV9-GFP or AAV9-PKP2 via retro-orbital injection.

### Whole-cell patch-clamp analysis

Whole-cell patch-clamp analysis of adult cardiomyocytes was performed as previously described, with some modifications^46,47^. In brief, single ventricular myocytes were isolated from mouse ventricles using the enzymatic digestion method by Langendorff. The single-pipette, whole-cell, voltage-clamp technique was performed by using a patch clamp amplifier (Axopatch 200B, Axon) to record membrane currents, and all experiments were performed at a room temperature of approximately 22 °C. The sodium channel current was recorded by stepped voltage-clamp (200 ms) between −80 and 15 mV in 5-mV steps from a holding potential of −80 mV, generated by a digital-to-analog converter (DigiData 1440, Axon) controlled by pCLAMP software (version 10.3, Axon). The modified Tyrode solution for perfusion contained 20 mM of NaCl (Sigma-Aldrich, 7683), 1 mM of MgCl_2_ (Sigma-Aldrich, M8266), 1 mM of CaCl_2_ (Sigma-Aldrich, C5670), 0.1 mM of CdCl_2_ (Sigma-Aldrich, 655198), 117.5 mM of CsCl (Sigma-Aldrich, 20966), 20 mM of HEPES (Sigma-Aldrich, 54457), 11 mM of glucose (Sigma-Aldrich, 7528) and 2 mM of nisoldipine (Sigma-Aldrich, N0165) with a pH of 7.4. The pipette solution contained 110 mM of CsCl (Sigma-Aldrich, 20966), 20 mM of EGTA (Sigma-Aldrich, 324626), 10 mM of TEA (Sigma-Aldrich, 86614), 5 mM of MgATP (Sigma-Aldrich, A9187) and 5 mM of HEPES (Sigma-Aldrich, 54457) with a pH of 7.2. Junction potentials were zeroed before formation of the membrane–pipette seal. Several minutes after seal formation, the membrane was ruptured by gentle suction to establish the whole-cell configuration for voltage clamping. Cell capacitance was measured by integrating the capacitive transient evoked by applying a 5-mV depolarizing step from a holding potential of −80 mV. Pipettes (resistance 3–5 MΩ) were pulled by a micropipette puller (Model P-87, Sutter Instrument).

### Mouse serum analysis assays

For liver enzyme analysis, serum was collected from mice at 6 months post AAV-PKP2 treatment, and serum ALP and ALT levels were assessed using a VetScan2 chemistry analyzer (Zoetis). For cytokine analysis, mouse serum was collected from late-stage AAV-administration mouse cohorts and subjected to cytokine profile assays as previously described^31^ and following the manufacturer’s instructions (R&D Systems, ARY028). In brief, cytokine array membranes were blocked for 1 h at room temperature and subjected to overnight incubation at 4 °C with 200 µl of mouse serum, followed by antibody detection cocktail incubation at room temperature for 1 h and then streptavidin–horseradish peroxidase (1:2,000, ThermoFisher Scientific, A16011) incubation for 30 min at room temperature. Cytokine signals on arrays were detected using the Chemiluminescence Reagent Mix according to the manufacturer’s instructions.

### Statistical analysis

All data are presented as mean ± s.e.m. GraphPad Prism was used for analyses, and significance was evaluated using Student’s two tailed *t*-test and one-way or two-way analysis of variance (ANOVA) with Tukey’s post-hoc multiple comparison tests. For Kaplan–Meier survival analysis, significance was assessed by the log-rank test. Categorical data was analyzed using Fisher’s exact test in RStudio software. A *P* value of <0.05 was considered statistically significant.

### Reporting summary

Further information on research design is available in the Nature Portfolio Reporting Summary linked to this article.

## Supplementary information

## Source data

## Figures and Tables

**Extended Data Table 1:**
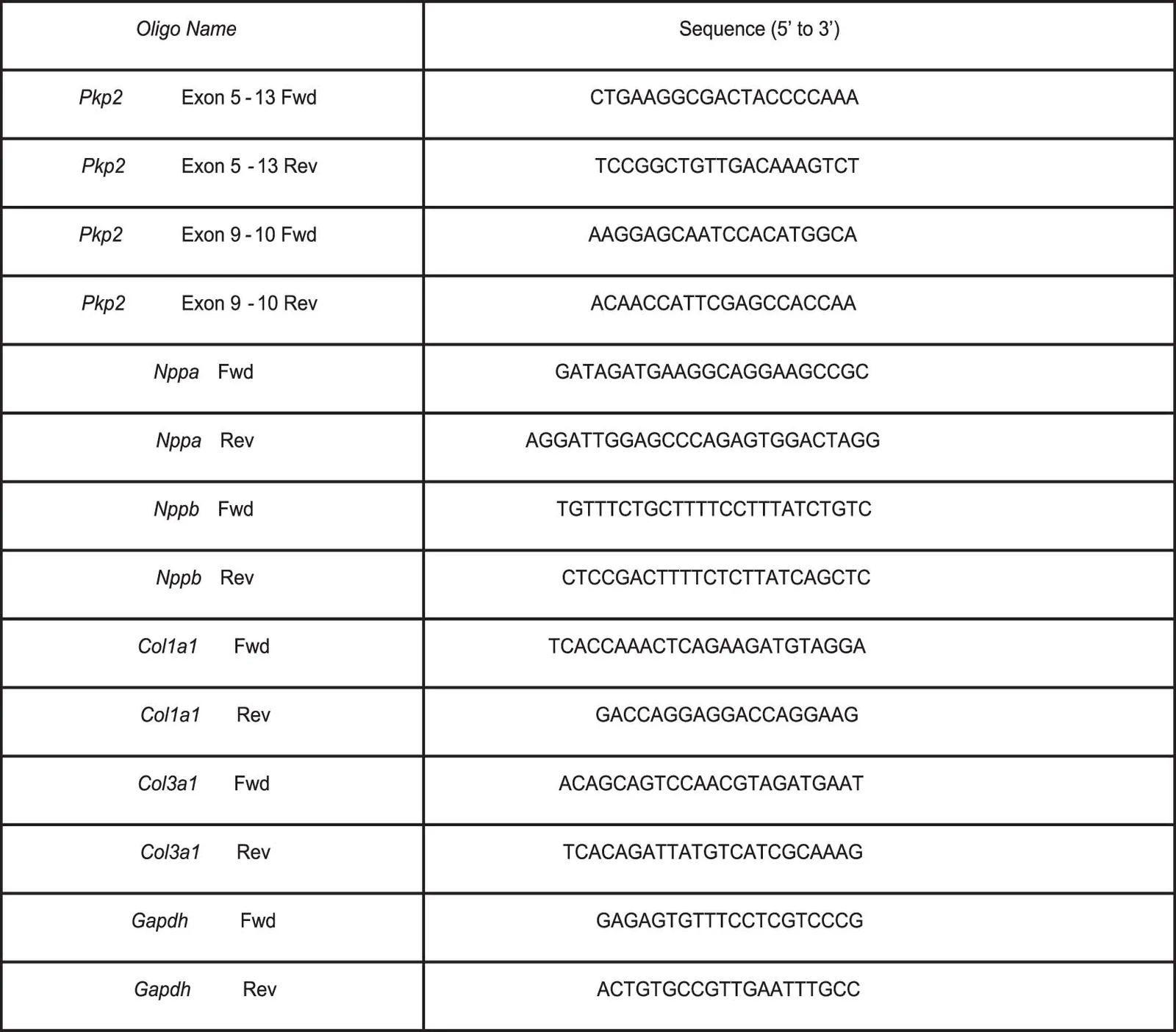
Primer sequences for real-time PCR analyses of PKP2 RNA as well as cardiac stress and fibrosis markers

## Data Availability

All data supporting the findings in this study are available within the paper and associated files. Source data are provided with this manuscript.

## References

[1] Thiene, G., Corrado, D. & Basso, C. Arrhythmogenic right ventricular cardiomyopathy/dysplasia. *Orphanet J. Rare Dis.* **2**, 45 (2007). 18001465 10.1186/1750-1172-2-45 PMC2222049

[2] Sen-Chowdhry, S. et al. Arrhythmogenic cardiomyopathy: etiology, diagnosis, and treatment. *Annu. Rev. Med.* **61**, 233–253 (2010). 20059337 10.1146/annurev.med.052208.130419

[3] Mattesi, G. et al. Natural history of arrhythmogenic cardiomyopathy. *J. Clin. Med.* **9**, 878 (2020). 32210158 10.3390/jcm9030878 PMC7141540

[4] Peters, S., Trummel, M. & Meyners, W. Prevalence of right ventricular dysplasia-cardiomyopathy in a non-referral hospital. *Int. J. Cardiol.* **97**, 499–501 (2004). 15561339 10.1016/j.ijcard.2003.10.037

[5] Elias Neto, J. et al. Arrhythmogenic right ventricular cardiomyopathy/dysplasia (ARVC/D) - what we have learned after 40 years of the diagnosis of this clinical entity. *Arq. Bras. Cardiol.* **112**, 91–103 (2019). 30673021 10.5935/abc.20180266 PMC6317628

[6] Idris, A., Shah, S. R. & Park, K. Right ventricular dysplasia: management and treatment in light of current evidence. *J. Community Hosp. Intern. Med. Perspect.* **8**, 101–106 (2018). 29915644 10.1080/20009666.2018.1472513 PMC5998293

[7] Wang, W., James, C. A. & Calkins, H. Diagnostic and therapeutic strategies for arrhythmogenic right ventricular dysplasia/cardiomyopathy patient. *Europace* **21**, 9–21 (2019). 29688316 10.1093/europace/euy063 PMC6321962

[8] Marcus, F. I., Edson, S. & Towbin, J. A. Genetics of arrhythmogenic right ventricular cardiomyopathy: a practical guide for physicians. *J. Am. Coll. Cardiol.* **61**, 1945–1948 (2013). 23500315 10.1016/j.jacc.2013.01.073

[9] Sheikh, F., Ross, R. S. & Chen, J. Cell-cell connection to cardiac disease. *Trends Cardiovasc. Med.* **19**, 182–190 (2009). 20211433 10.1016/j.tcm.2009.12.001 PMC3601820

[10] Rampazzo, A. et al. Intercalated discs and arrhythmogenic cardiomyopathy. *Circ. Cardiovasc. Genet.* **7**, 930–940 (2014). 25516623 10.1161/CIRCGENETICS.114.000645

[11] Gerull, B. et al. Mutations in the desmosomal protein plakophilin-2 are common in arrhythmogenic right ventricular cardiomyopathy. *Nat. Genet.* **36**, 1162–1164 (2004). 15489853 10.1038/ng1461

[12] Kirchner, F. et al. Molecular insights into arrhythmogenic right ventricular cardiomyopathy caused by plakophilin-2 missense mutations. *Circ. Cardiovasc. Genet.* **5**, 400–411 (2012). 22781308 10.1161/CIRCGENETICS.111.961854

[13] Rasmussen, T. B. et al. Truncating plakophilin-2 mutations in arrhythmogenic cardiomyopathy are associated with protein haploinsufficiency in both myocardium and epidermis. *Circ. Cardiovasc. Genet.* **7**, 230–240 (2014). 24704780 10.1161/CIRCGENETICS.113.000338

[14] Akdis, D. et al. Myocardial expression profiles of candidate molecules in patients with arrhythmogenic right ventricular cardiomyopathy/dysplasia compared to those with dilated cardiomyopathy and healthy controls. *Heart Rhythm* **13**, 731–741 (2016). 26569459 10.1016/j.hrthm.2015.11.010

[15] Groeneweg, J. A. et al. Functional assessment of potential splice site variants in arrhythmogenic right ventricular dysplasia/cardiomyopathy. *Heart Rhythm* **11**, 2010–2017 (2014). 25087486 10.1016/j.hrthm.2014.07.041

[16] Lim, K. H. et al. Using positional distribution to identify splicing elements and predict pre-mRNA processing defects in human genes. *Proc. Natl Acad. Sci. USA* **108**, 11093–11098 (2011). 21685335 10.1073/pnas.1101135108 PMC3131313

[17] Anna, A. & Monika, G. Splicing mutations in human genetic disorders: examples, detection, and confirmation. *J. Appl. Genet.* **59**, 253–268 (2018). 29680930 10.1007/s13353-018-0444-7 PMC6060985

[18] Attali, R. et al. Mutation of SYNE-1, encoding an essential component of the nuclear lamina, is responsible for autosomal recessive arthrogryposis. *Hum. Mol. Genet.* **18**, 3462–3469 (2009). 19542096 10.1093/hmg/ddp290

[19] Guernsey, D. L. et al. Mutation in the gene encoding ubiquitin ligase LRSAM1 in patients with Charcot-Marie-Tooth disease. *PLoS Genet.* **6**, e1001081 (2010). 20865121 10.1371/journal.pgen.1001081 PMC2928813

[20] Watanabe, T. et al. *A* mutant mRNA expression in an endomyocardial biopsy sample obtained from a patient with a cardiac variant of Fabry disease caused by a novel acceptor splice site mutation in the invariant AG of intron 5 of the alpha-galactosidase A gene. *Intern. Med.* **52**, 777–780 (2013). 23545674 10.2169/internalmedicine.52.9213

[21] Svensson, A. et al. Arrhythmogenic right ventricular cardiomyopathy - 4 Swedish families with an associated PKP2 c.2146-1G>C variant. *Am. J. Cardiovasc. Dis.* **6**, 55–65 (2016). 27335691 PMC4913215

[22] Syrris, P. et al. Clinical expression of plakophilin-2 mutations in familial arrhythmogenic right ventricular cardiomyopathy. *Circulation* **113**, 356–364 (2006). 16415378 10.1161/CIRCULATIONAHA.105.561654

[23] Walsh, R. et al. Reassessment of Mendelian gene pathogenicity using 7,855 cardiomyopathy cases and 60,706 reference samples. *Genet. Med.* **19**, 192–203 (2017). 27532257 10.1038/gim.2016.90 PMC5116235

[24] Lyon, R. C. et al. Connexin defects underlie arrhythmogenic right ventricular cardiomyopathy in a novel mouse model. *Hum. Mol. Genet.* **23**, 1134–1150 (2014). 24108106 10.1093/hmg/ddt508 PMC3919010

[25] Asimaki, A., Kleber, A. G. & Saffitz, J. E. Pathogenesis of arrhythmogenic cardiomyopathy. *Can. J. Cardiol.* **31**, 1313–1324 (2015). 26199027 10.1016/j.cjca.2015.04.012 PMC4619183

[26] Cerrone, M. & Delmar, M. Desmosomes and the sodium channel complex: implications for arrhythmogenic cardiomyopathy and Brugada syndrome. *Trends Cardiovasc. Med.* **24**, 184–190 (2014). 24656989 10.1016/j.tcm.2014.02.001 PMC4099253

[27] Basso, C. et al. Ultrastructural evidence of intercalated disc remodelling in arrhythmogenic right ventricular cardiomyopathy: an electron microscopy investigation on endomyocardial biopsies. *Eur. Heart J.* **27**, 1847–1854 (2006). 16774985 10.1093/eurheartj/ehl095

[28] Liang, Y. et al. Desmosomal COP9 regulates proteome degradation in arrhythmogenic right ventricular dysplasia/cardiomyopathy. *J. Clin. Invest.* **131**, e137689 (2021). 33857019 10.1172/JCI137689 PMC8159691

[29] Basso, C. & Thiene, G. Adipositas cordis, fatty infiltration of the right ventricle, and arrhythmogenic right ventricular cardiomyopathy. Just a matter of fat?. *Cardiovasc. Pathol.* **14**, 37–41 (2005). 15710290 10.1016/j.carpath.2004.12.001

[30] Te Riele, A. S. et al. Mutation-positive arrhythmogenic right ventricular dysplasia/cardiomyopathy: the triangle of dysplasia displaced. *J. Cardiovasc. Electrophysiol.* **24**, 1311–1320 (2013). 23889974 10.1111/jce.12222 PMC3971054

[31] Chelko, S. P. et al. Therapeutic modulation of the immune response in arrhythmogenic cardiomyopathy. *Circulation* **140**, 1491–1505 (2019). 31533459 10.1161/CIRCULATIONAHA.119.040676 PMC6817418

[32] Bezzerides, V. J. et al. Gene therapy for catecholaminergic polymorphic ventricular tachycardia by inhibition of Ca(2+)/calmodulin-dependent kinase II. *Circulation* **140**, 405–419 (2019). 31155924 10.1161/CIRCULATIONAHA.118.038514 PMC7274838

[33] Lambert, J. M. et al. Mechanisms and regulation of nonsense-mediated mRNA decay and nonsense-associated altered splicing in lymphocytes. *Int. J. Mol. Sci.* **21**, 1335 (2020). 32079193 10.3390/ijms21041335 PMC7072976

[34] Grossmann, K. S. et al. Requirement of plakophilin 2 for heart morphogenesis and cardiac junction formation. *J. Cell Biol.* **167**, 149–160 (2004). 15479741 10.1083/jcb.200402096 PMC2172504

[35] Moncayo-Arlandi, J. et al. Molecular disturbance underlies to arrhythmogenic cardiomyopathy induced by transgene content, age and exercise in a truncated PKP2 mouse model. *Hum. Mol. Genet.* **25**, 3676–3688 (2016). 27412010 10.1093/hmg/ddw213

[36] Cruz, F. M. et al. Exercise triggers ARVC phenotype in mice expressing a disease-causing mutated version of human plakophilin-2. *J. Am. Coll. Cardiol.* **65**, 1438–1450 (2015). 25857910 10.1016/j.jacc.2015.01.045

[37] McKoy, G. et al. Identification of a deletion in plakoglobin in arrhythmogenic right ventricular cardiomyopathy with palmoplantar keratoderma and woolly hair (Naxos disease). *Lancet* **355**, 2119–2124 (2000). 10902626 10.1016/S0140-6736(00)02379-5

[38] Zhang, Z. et al. Normalization of Naxos plakoglobin levels restores cardiac function in mice. *J. Clin. Invest.* **125**, 1708–1712 (2015). 25705887 10.1172/JCI80335 PMC4396479

[39] Cerrone, M. et al. Plakophilin-2 is required for transcription of genes that control calcium cycling and cardiac rhythm. *Nat. Commun.* **8**, 106 (2017). 28740174 10.1038/s41467-017-00127-0 PMC5524637

[40] Delmar, M. & McKenna, W. J. The cardiac desmosome and arrhythmogenic cardiomyopathies: from gene to disease. *Circ. Res.* **107**, 700–714 (2010). 20847325 10.1161/CIRCRESAHA.110.223412

[41] Lyon, R. C. et al. Mechanotransduction in cardiac hypertrophy and failure. *Circ. Res.* **116**, 1462–1476 (2015). 25858069 10.1161/CIRCRESAHA.116.304937 PMC4394185

[42] Chen, X. et al. Protein binding and functional characterization of plakophilin 2. Evidence for its diverse roles in desmosomes and beta-catenin signaling. *J. Biol. Chem.* **277**, 10512–10522 (2002). 11790773 10.1074/jbc.M108765200

[43] Bass-Zubek, A. E. et al. Plakophilins: multifunctional scaffolds for adhesion and signaling. *Curr. Opin. Cell Biol.* **21**, 708–716 (2009). 19674883 10.1016/j.ceb.2009.07.002 PMC3091506

[44] Ma, X. et al. CRISPR/Cas9-mediated gene manipulation to create single-amino-acid-substituted and floxed mice with a cloning-free method. *Sci. Rep.* **7**, 42244 (2017). 28176880 10.1038/srep42244 PMC5296764

[45] Bravo-Hernandez, M. et al. Spinal subpial delivery of AAV9 enables widespread gene silencing and blocks motoneuron degeneration in ALS. *Nat. Med.* **26**, 118–130 (2020). 31873312 10.1038/s41591-019-0674-1 PMC8171115

[46] Chiang, C. E., Wang, T. M. & Luk, H. N. Inhibition of L-type Ca^2+^ current in guinea pig ventricular myocytes by cisapride. *J. Biomed. Sci.* **11**, 303–314 (2004). 15067213 10.1007/BF02254434

[47] Sato, P. Y. et al. Loss of plakophilin-2 expression leads to decreased sodium current and slower conduction velocity in cultured cardiac myocytes. *Circ. Res.* **105**, 523–526 (2009). 19661460 10.1161/CIRCRESAHA.109.201418 PMC2742576

