# Supplementary material for: Plakophilin 2 gene therapy prevents and rescues arrhythmogenic right ventricular cardiomyopathy in a mouse model harboring patient genetics
Source: Nat Cardiovasc Res. 2023 Dec 7;2(12):1246–61. doi: 10.1038/s44161-023-00370-3 (PMC11357983; doi:10.1038/s44161-023-00370-3)
Supplement: Supplementary file 1 — Supplementary Table 1 [file 44161_2023_370_MOESM1_ESM.pdf]

nature cardiovascular research

Article https://doi.org/10.1038/s44161-023-00370-3

Plakophilin 2 gene therapy prevents and rescues arrhythmogenic right ventricular cardiomyopathy in a mouse model harboring patient genetics

In the format provided by the authors and unedited

Supplemental Tables

	Ctrl	Het	Hom
Number	33	79	30
Actual Percentage	23%	56%	21%
Expected Percentage	25%	50%	25%

Supplementary Table 1. Offspring from PKP2 heterozygous mutant breeding strategy were viable and born at Mendelian ratios.
