# Supplementary figures and images for: Plakophilin 2 gene therapy prevents and rescues arrhythmogenic right ventricular cardiomyopathy in a mouse model harboring patient genetics
Source: Nat Cardiovasc Res. 2023 Dec 7;2(12):1246–61. doi: 10.1038/s44161-023-00370-3 (PMC11357983; doi:10.1038/s44161-023-00370-3)

Unedited Figure 3 c

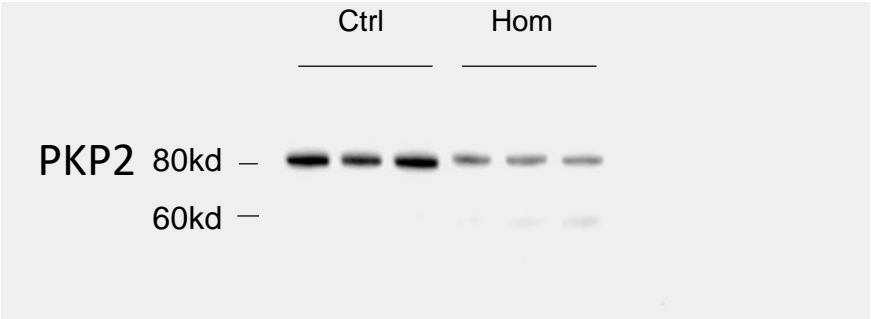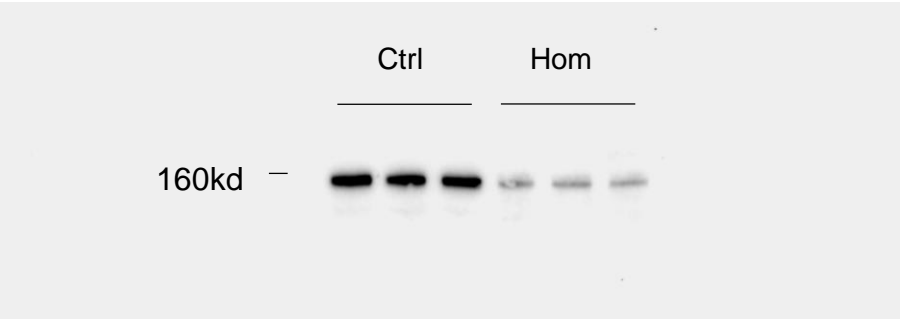

DSG2

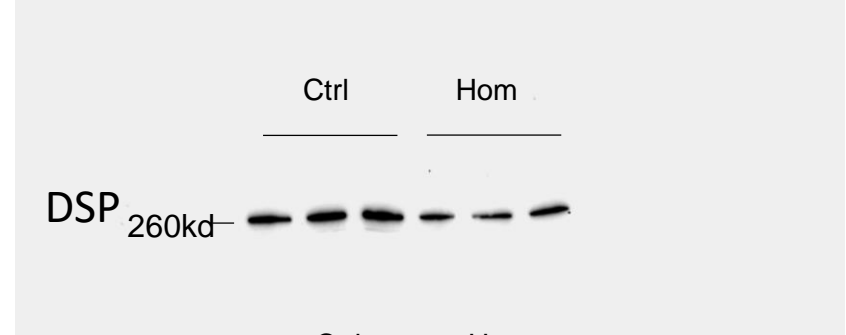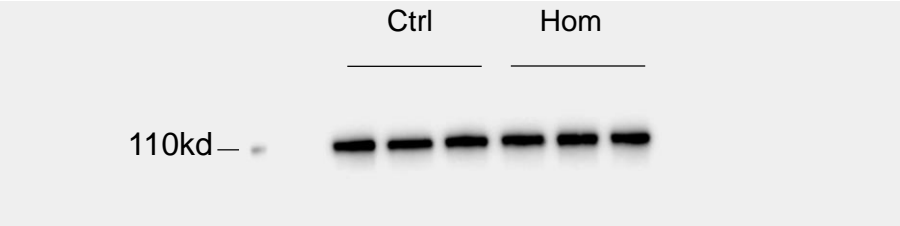

N-cad

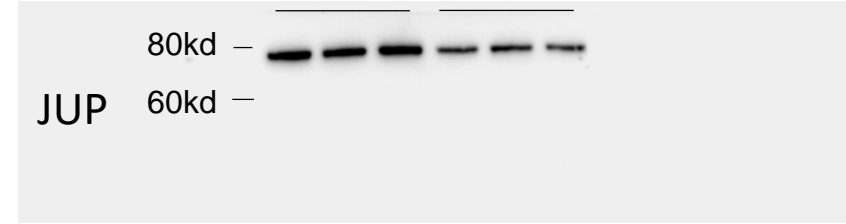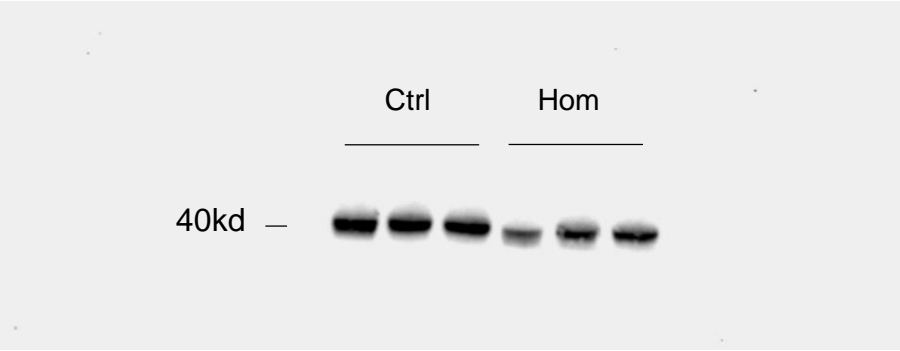

CX43

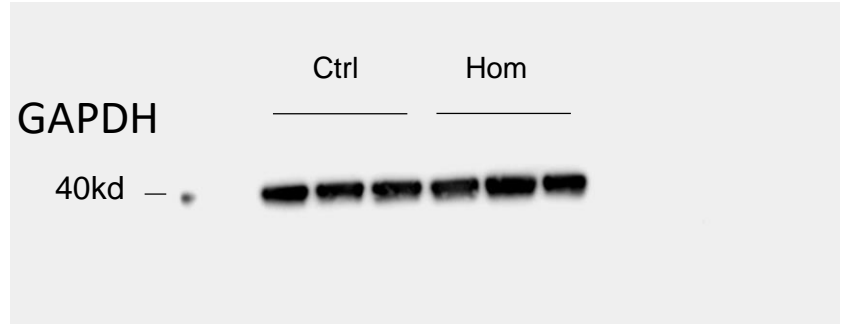

Supplement: Supplementary file 3 — Unprocessed western blots [file 44161_2023_370_MOESM3_ESM.pdf]

Unedited Figure 4 a

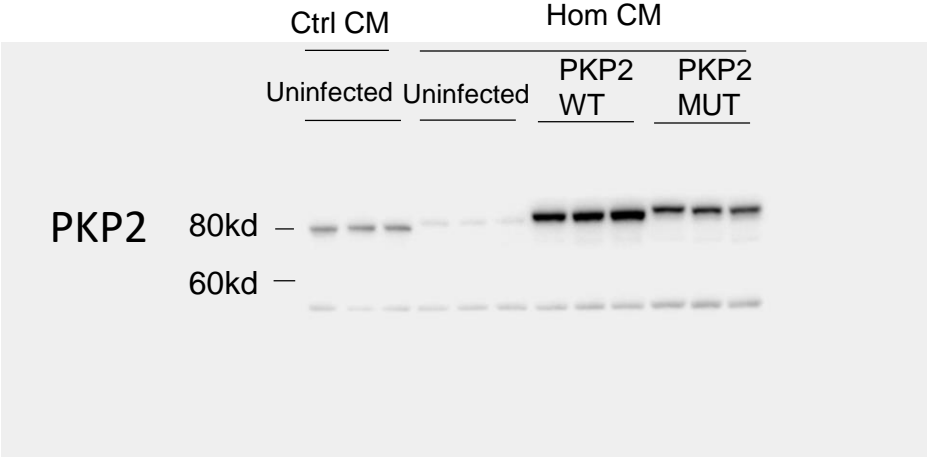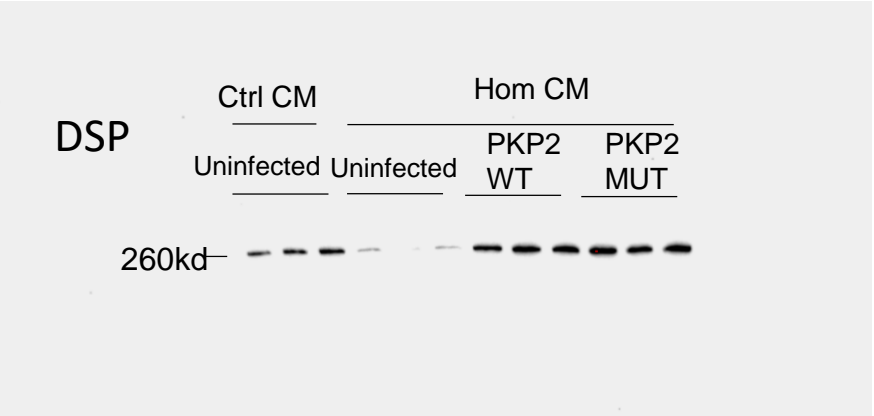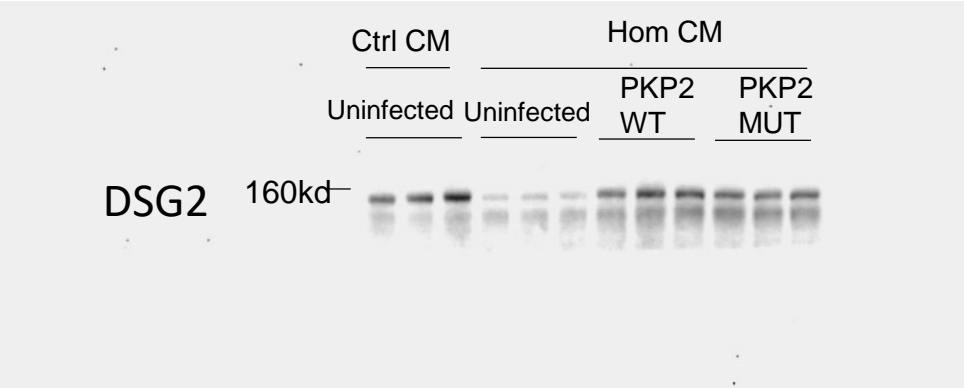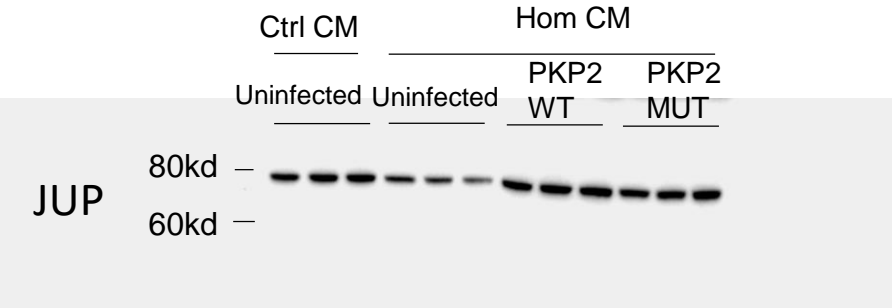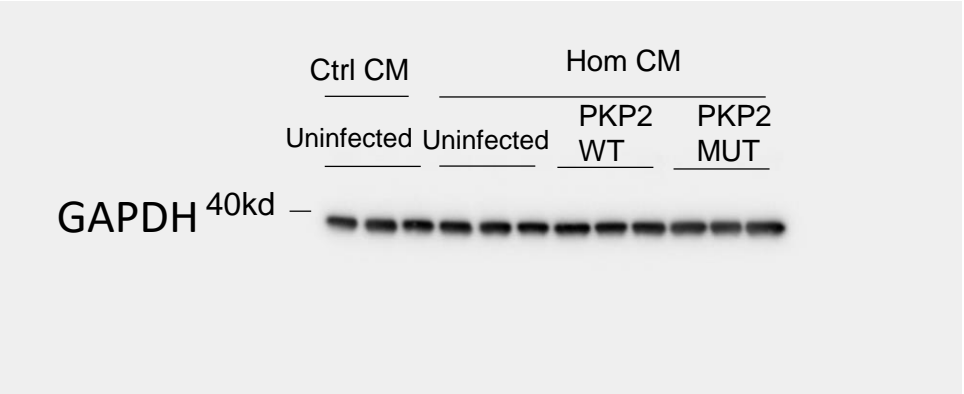

Supplement: Supplementary file 4 — Unprocessed western blots [file 44161_2023_370_MOESM4_ESM.pdf]

Unedited Figure 5d

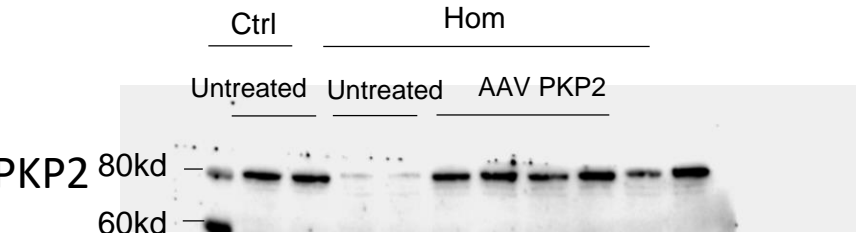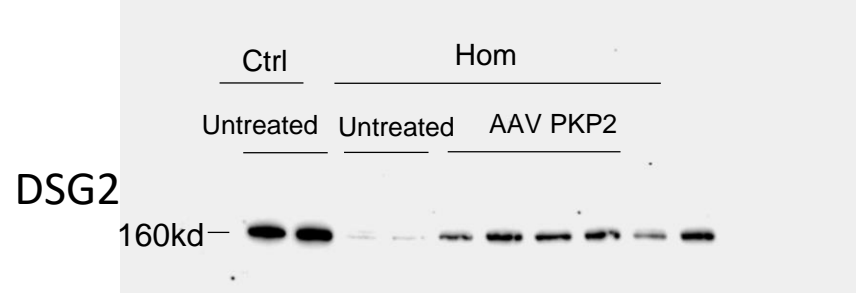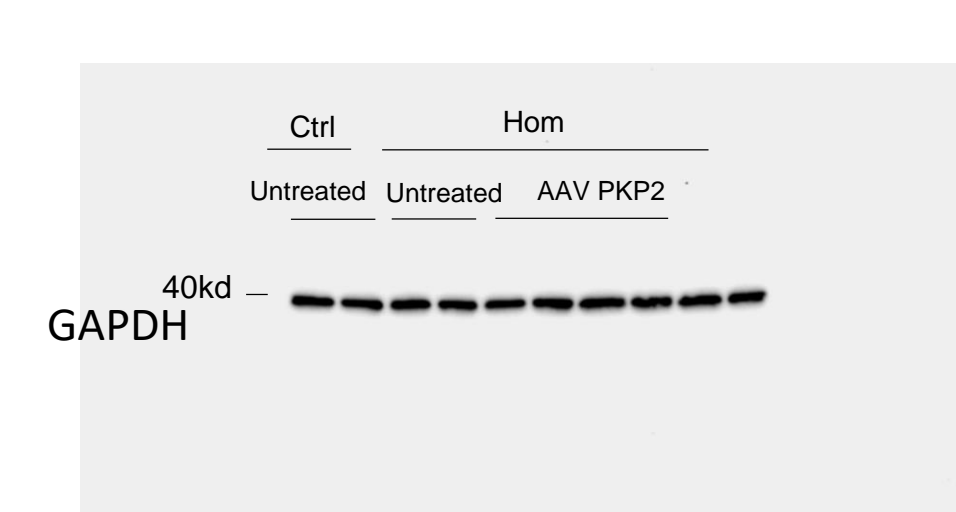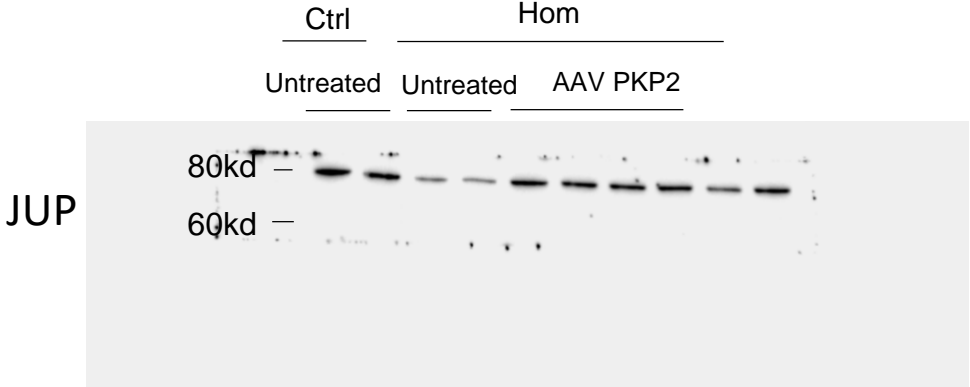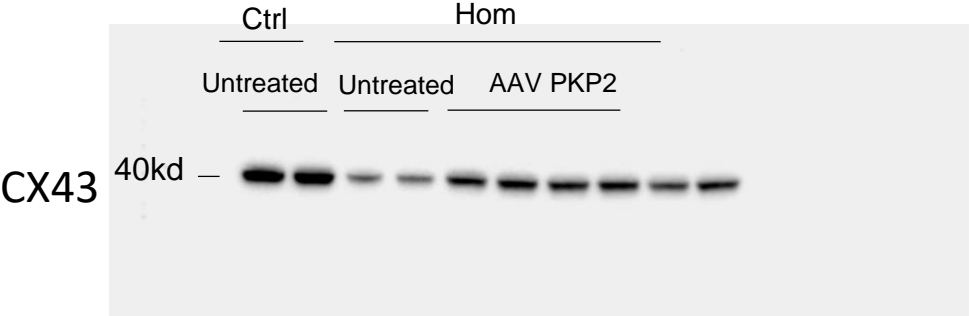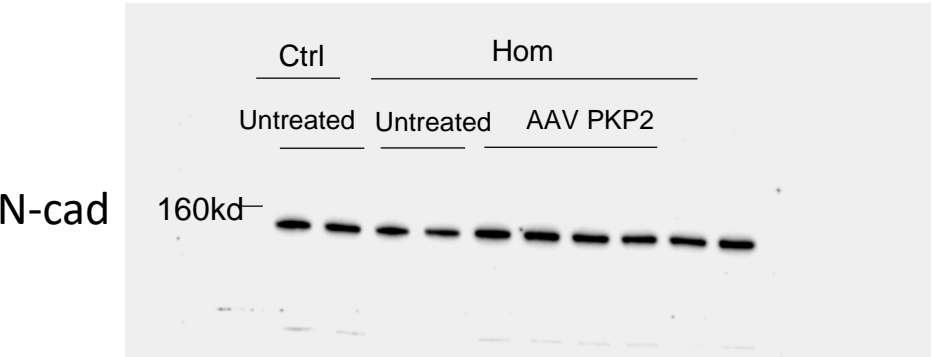

Supplement: Supplementary file 5 — Unprocessed western blots [file 44161_2023_370_MOESM5_ESM.pdf]

Unedited Figure 7c

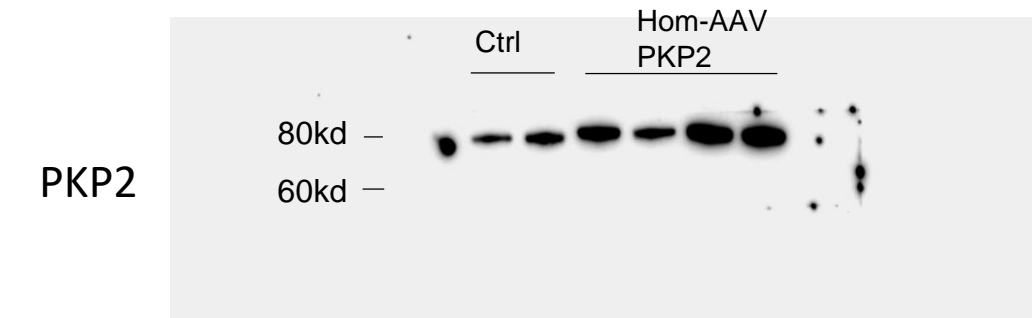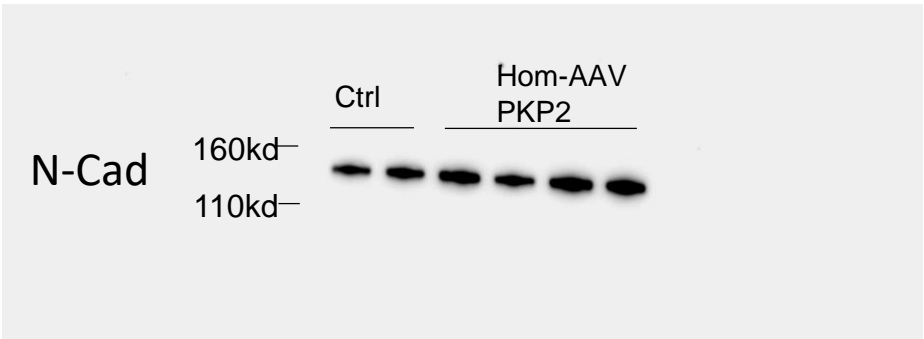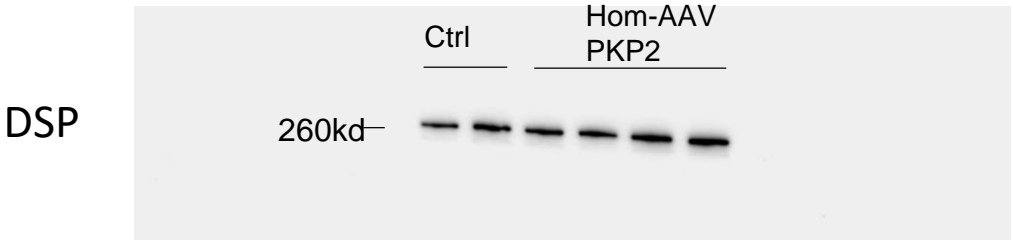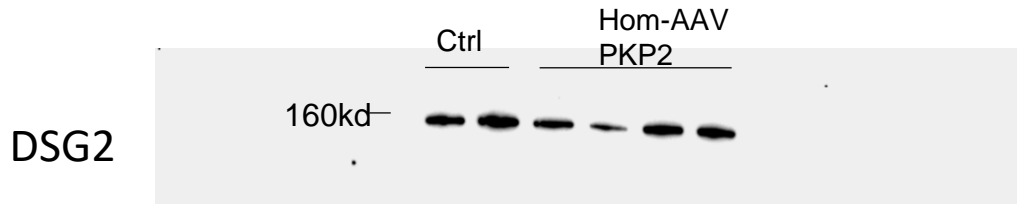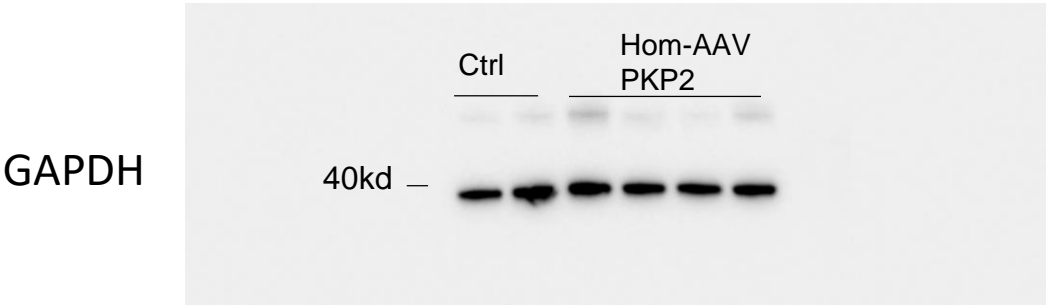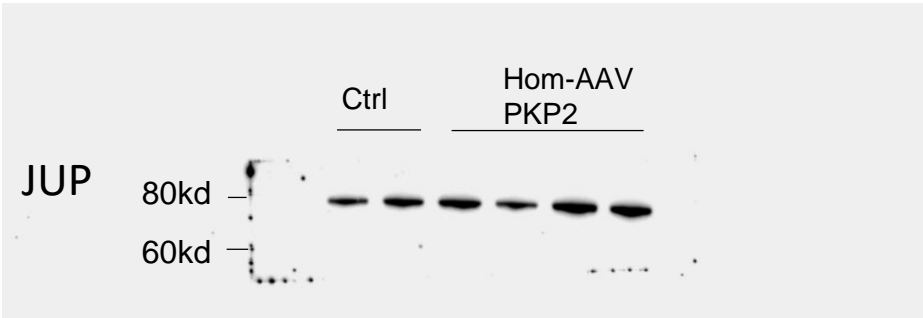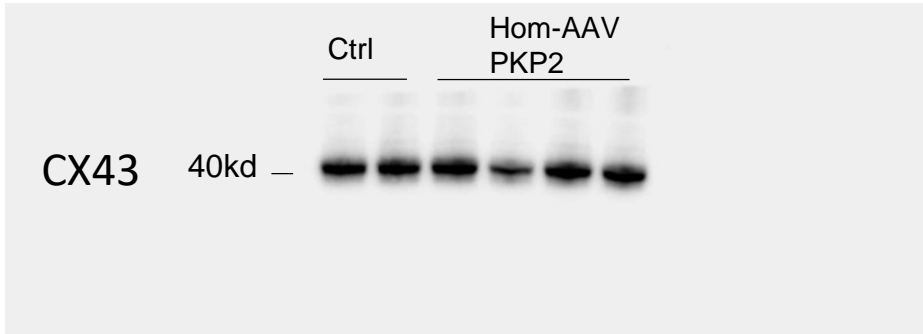

Supplement: Supplementary file 6 — Unprocessed western blots [file 44161_2023_370_MOESM6_ESM.pdf]

Unedited Figure 8b

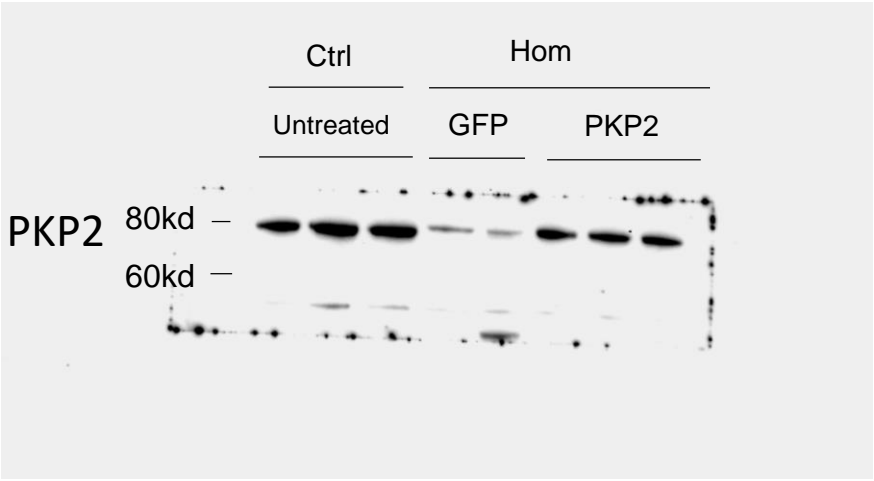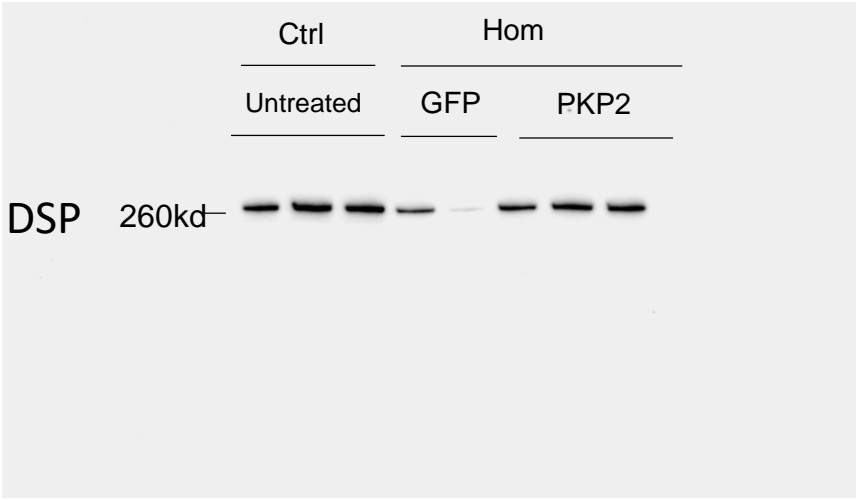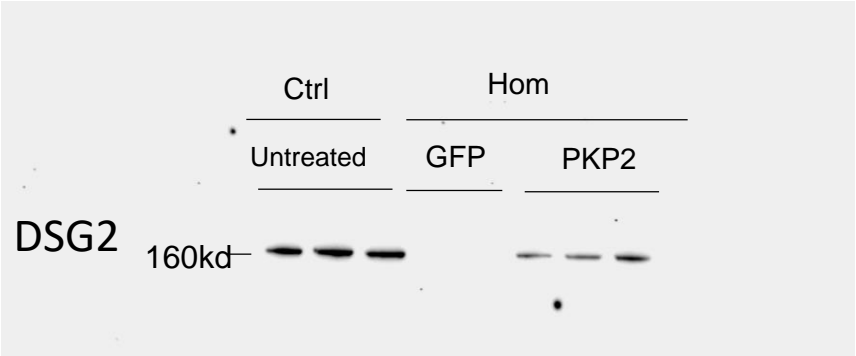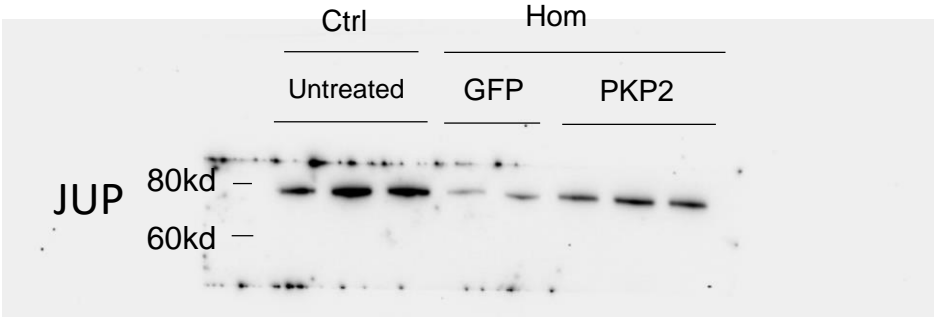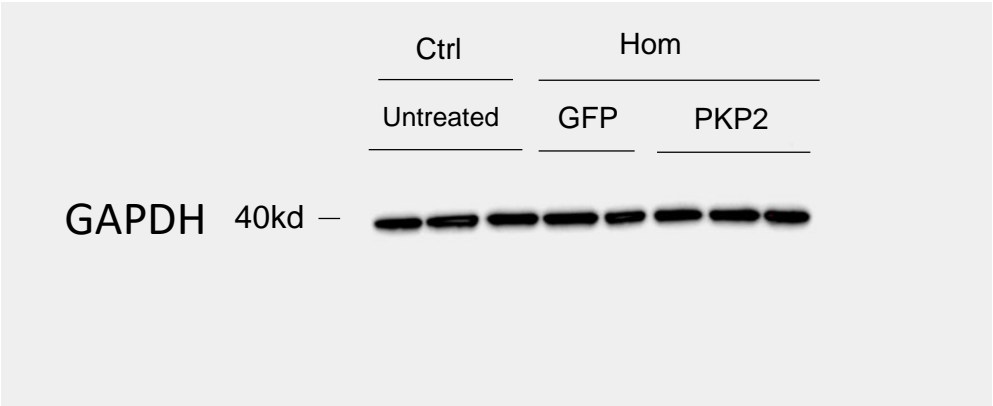

Supplement: Supplementary file 7 — Unprocessed western blots [file 44161_2023_370_MOESM7_ESM.pdf]

Unedited Extended Data 3 b

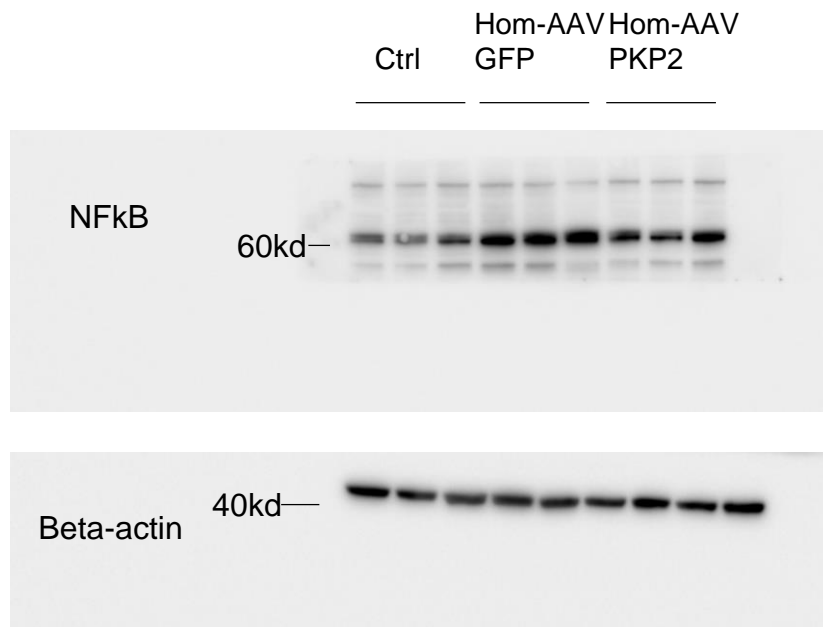

Supplement: Supplementary file 8 — Unprocessed western blots [file 44161_2023_370_MOESM8_ESM.pdf]

Unedited Extended Data 5 d

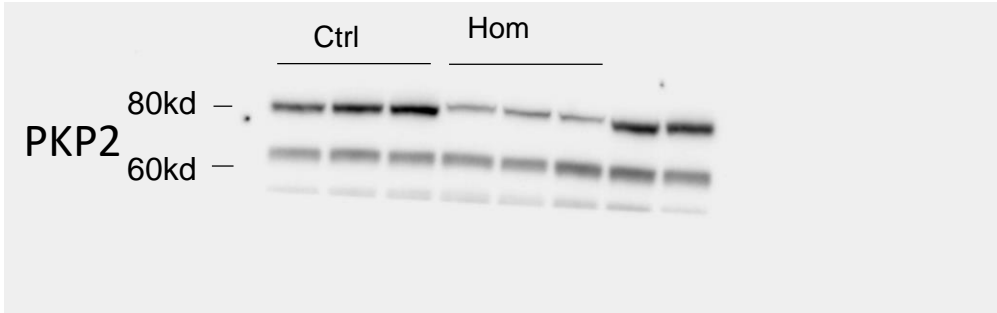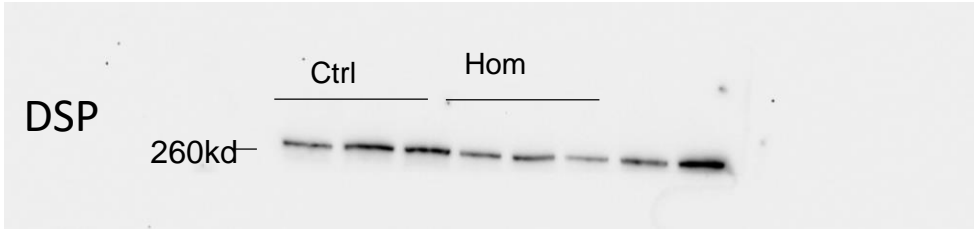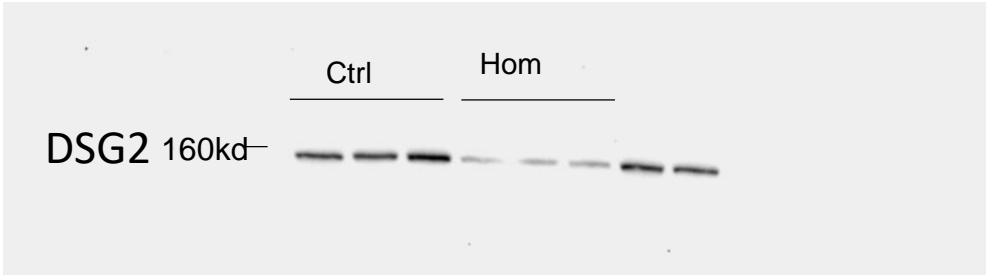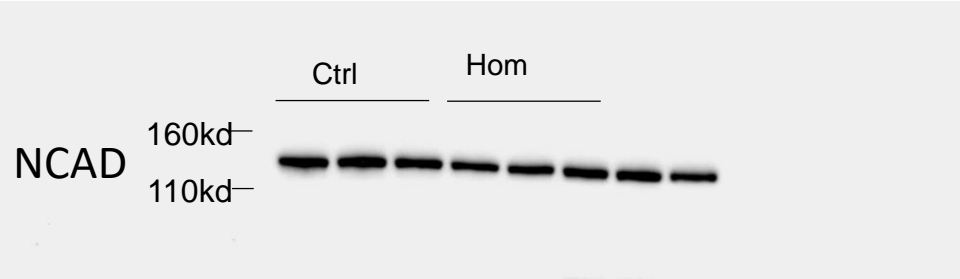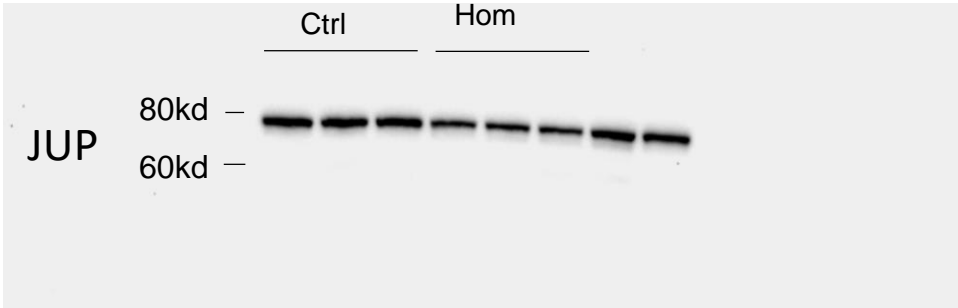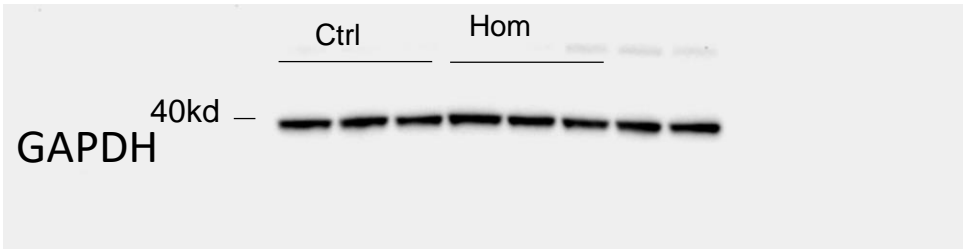

Supplement: Supplementary file 9 — Unprocessed western blots [file 44161_2023_370_MOESM9_ESM.pdf]

Unedited Extended Data 6 a

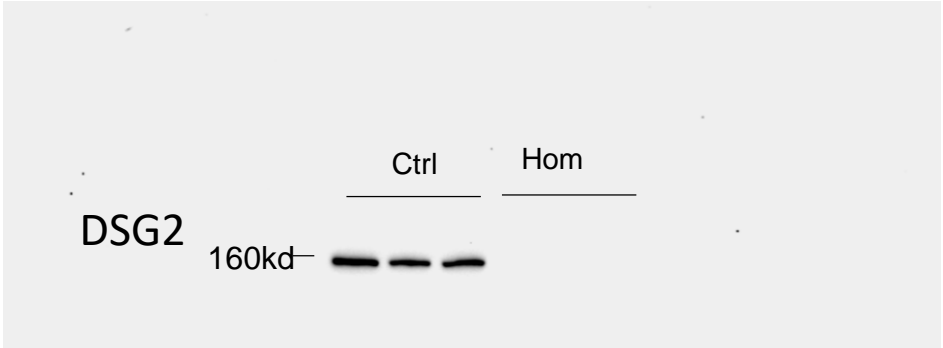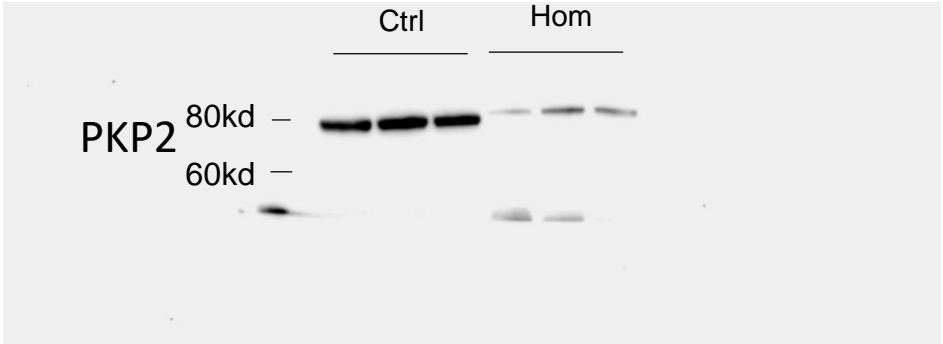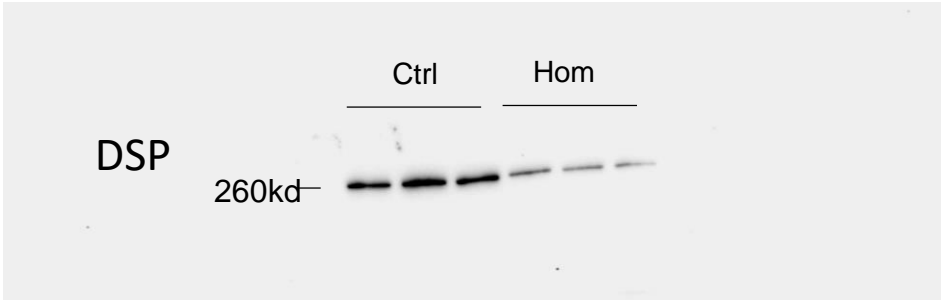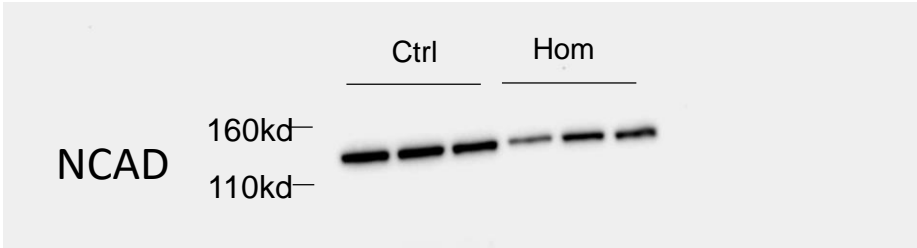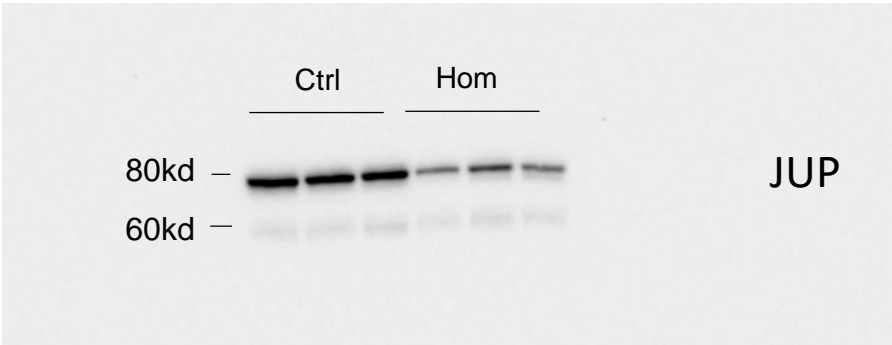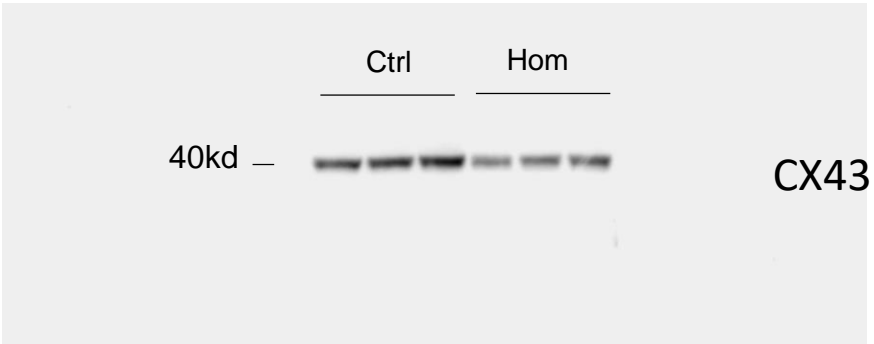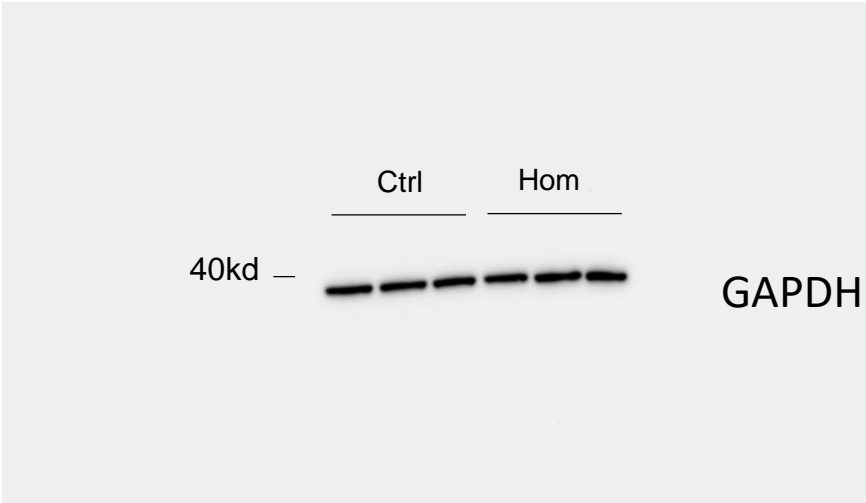

Supplement: Supplementary file 10 — Unprocessed western blots [file 44161_2023_370_MOESM10_ESM.pdf]

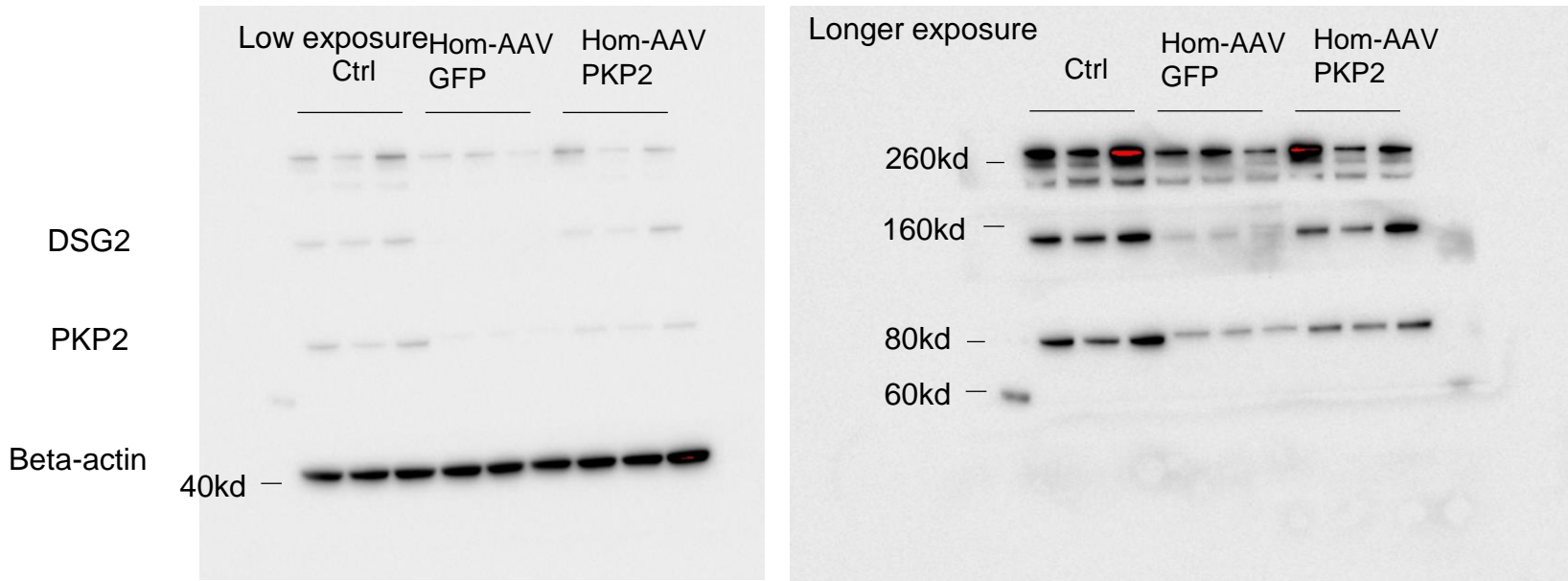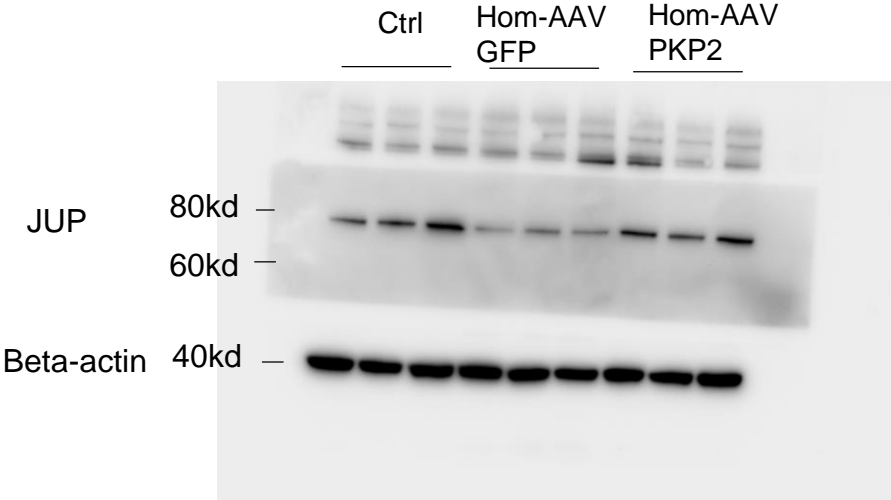

Supplement: Supplementary file 11 — Unprocessed western blots [file 44161_2023_370_MOESM11_ESM.pdf]
